# Alteration of Gene Expression, DNA Methylation, and Histone Methylation in Free Radical Scavenging Networks in Adult Mouse Hippocampus following Fetal Alcohol Exposure

**DOI:** 10.1371/journal.pone.0154836

**Published:** 2016-05-02

**Authors:** Eric J. Chater-Diehl, Benjamin I. Laufer, Christina A. Castellani, Bonnie L. Alberry, Shiva M. Singh

**Affiliations:** Molecular Genetics Unit, Department of Biology, Western University, London, Ontario, Canada; Chung-Ang University, REPUBLIC OF KOREA

## Abstract

The molecular basis of Fetal Alcohol Spectrum Disorders (FASD) is poorly understood; however, epigenetic and gene expression changes have been implicated. We have developed a mouse model of FASD characterized by learning and memory impairment and persistent gene expression changes. Epigenetic marks may maintain expression changes over a mouse’s lifetime, an area few have explored. Here, mice were injected with saline or ethanol on postnatal days four and seven. At 70 days of age gene expression microarray, methylated DNA immunoprecipitation microarray, H3K4me3 and H3K27me3 chromatin immunoprecipitation microarray were performed. Following extensive pathway analysis of the affected genes, we identified the top affected gene expression pathway as “Free radical scavenging”. We confirmed six of these changes by droplet digital PCR including the caspase *Casp3* and Wnt transcription factor *Tcf7l2*. The top pathway for all methylation-affected genes was “Peroxisome biogenesis”; we confirmed differential DNA methylation in the *Acca1* thiolase promoter. Altered methylation and gene expression in oxidative stress pathways in the adult hippocampus suggests a novel interface between epigenetic and oxidative stress mechanisms in FASD.

## Introduction

Fetal alcohol spectrum disorder (FASD) refers to the neurological, developmental, and behavioural abnormalities arising from *in utero* ethanol exposure. It is characterized by a range of behavioural aberrations including anxiety, depression, and impaired learning and memory that can persist to adulthood [1–3]. The short-term effects of ethanol on the brain are well characterized, including altered insulin signalling, apoptosis, and synaptic remodelling [4]. Long after ethanol exposure, alterations in gene expression in key brain regions have been identified by various researchers [5–8]. In particular, our group and others have focused on the hippocampus due to its central role in learning and memory. The long-term maintenance mechanism behind these expression changes and how they may underlie FASD-related phenotypes remain elusive.

Epigenetic marks are a strong candidate for maintaining FASD-related gene expression changes. Alcohol indirectly affects DNA and histone methylation by altering one-carbon metabolism from which methyl groups are derived [9,10]. Ethanol also reduces the activity of methionine synthase [11]. Both of these alterations decrease the availability of the methyl-donor S-adenosylmethionine (SAM). In the hippocampus, prenatal ethanol exposure leads to maintained expression changes in one-carbon metabolism genes [8]. Further, ethanol alters the redox state of the cell which can affect methylation pathways [12].

A number of studies have reported ethanol-induced alterations in DNA methylation [13–15]. In the hippocampus specifically, prenatal ethanol exposure has been associated with changes in DNA methylation and its binding proteins [16]. Additionally, global hypermethylation in the hippocampus occurs in young rats exposed to ethanol during synaptogenesis [17]. To date, no study has performed a high-resolution genome-wide analysis of DNA methylation in the ethanol-exposed hippocampus.

Many lines of evidence also suggest a role of histone methylation in FASD. First, histone methylation is far more liable and environmentally responsive than DNA methylation [18]. Second, ethanol causes changes in histone methyltransferase abundance, contributing to ethanol-induced neurodegeneration during neonatal development in mice [19]. Finally, critical neurodevelopmental genes show alterations in histone H3 lysine 4 trimethylation (H3K4me3) and histone H3 lysine 27 trimethylation (H3K27me3) at their promoters in neuronal stem cells exposed to ethanol [20]. These studies strongly suggest ethanol may act in part through alteration of nuclear architecture via histone modifications. Specifically, changes in H3K4me3 and H3K27me3 are often the focus of environmental epigenetic studies due to their tight association with gene expression at promoters: H3K4me3 is a hallmark of active transcriptional start sites [21], while H3K27me3 is associated with inactive but not constitutively silenced genes [21]. To the best of our knowledge, the long-term effects of ethanol on H3K4me3 and H3K27me3 *in vivo* have not been previously assessed. To this end, we sought to undertake a comprehensive assessment of H3K4me3, H3K27me3 and DNA methylation changes in adult mice exposed to alcohol during development.

Here, we use a binge exposure model of FASD in which mice are injected with ethanol during the first postnatal week. This time period is neurodevelopmentally equivalent to the human third trimester and coincides with the peak of synaptogenesis [22]. Binge drinking patterns result in the highest blood alcohol concentrations (BAC) [23]. If these episodes coincide with critical neurodevelopmental events, there can be significant adverse effects to the fetus [24]. Binge drinking is also a pattern often reported by alcohol-consuming pregnant women [25]. The mouse hippocampus undergoes neuronal differentiation and synaptogenesis during the first postnatal week [26]. Ethanol exposure during this critical time affects nearly all levels of hippocampal structure and function [27]. The resulting synaptic changes affect long-term potentiation, a strong correlate of learning and memory [28]. Indeed, a number of behavioural abnormalities are reported including spatial learning and memory impairment [6], directly implicating hippocampal involvement [27]. Ethanol exposure at this time also causes changes in brain gene expression [22], DNA methylation, and non-coding RNA (ncRNA) expression [29]. As such, trimester three binge models are very common in genetic studies of FASD.

In this experiment, we assess gene/miRNA expression, DNA methylation, and histone modification changes in adult mouse hippocampus after neonatal ethanol exposure. Using chromatin immunoprecipitation (ChIP) with promoter microarray (ChIP-chip) we identified hundreds of changes in H3K4me3 and H3K27me3. Using methylated DNA immunoprecipitation (MeDIP) coupled to promoter microarray (MeDIP-chip) we identified numerous DNA methylation changes. Using Affymetrix gene expression microarrays we also identified numerous gene expression changes in the same mice. The genes of interest have low-fold changes and low expression levels, so we chose to confirm differential expression using droplet digital PCR (ddPCR) which is well suited for these conditions [30]. Some of the genes identified have both gene expression and DNA methylation changes, but more importantly many of the top affected pathways are related across data sets. The most highly affected genes and pathways are oxidative stress pathways which are highly relevant to FASD etiology [12].

## Results

### Gene expression analysis

**Fig 1:**
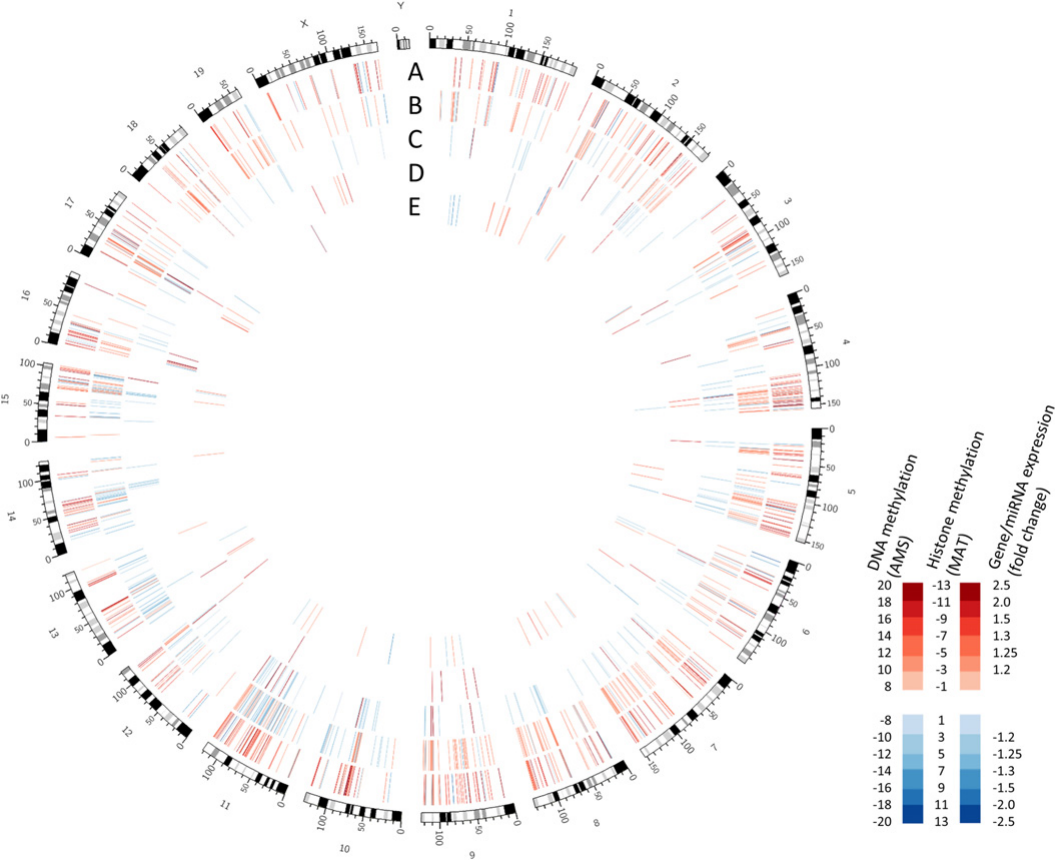
Global changes in DNA methylation, histone methylation, miRNA expression, and gene expression in adult mice in response to neonatal ethanol exposure. Tracks show alterations in: (A) DNA methylation as measured by absolute methylation score (AMS) *p*<0.001; (B) H3k27me3 and (C) H3k4me3 measured by model-based analysis of tiling arrays (MAT) score, *p*<0.001; (D) miRNA expression and (E) gene expression *p*<0.05, fold-change>1.2.

We identified 60 genes differentially expressed in ethanol exposed mouse hippocampus (Fig 1). Two thirds of these were upregulated, and one third down-regulated in response to ethanol (Table 1). 61 ncRNAs (37 of which were mature microRNAs) were also differentially expressed. Using IPA target filter, we identified five differentially expressed microRNAs (miRNAs) predicted to target four differentially expressed genes in reciprocal relationships (Table 2). Of the 60 differentially expressed genes, 8 (13.3%) showed a change in DNA methylation, 11 (18.3%) showed a change in H3K4me3, and 4 (6.7%). showed a change in H3K27me3 in their promoter sequence or gene body (Table 1).

**Table 1:** Differentially expressed genes in adult mouse hippocampus exposed to ethanol during development identified by gene expression microarray.

Gene expression			5mC DMR		H3K4me3 RDHM		H3K27me3 RDHM	
Gene Symbol	*p*-value	Fold change	AMS score	*p*-value	MAT score	*p*-value	MAT score	*p*-value
*Tcf7l2*	0.032	1.50	9.87	0.007	-3.12	0.004	1.86	0.003
					-4.38	0.000		
*Synpo2*	0.047	1.43	11.82	0.004	3.10	0.009		
*Vipr2*	0.043	1.42	10.32	0.002				
*Cypt2*	0.012	1.40						
*Defb5*	0.002	1.39						
*Serpinb1b*	0.027	1.35						
*Gm8994*	0.021	1.32			3.14	0.008		
*Gm7168*	0.016	1.31						
*Olfr119*	0.007	1.30			-2.93	0.008		
*Vmn2r15*	0.049	1.29					1.44	0.004
*Cfhr2*	0.023	1.29			3.09	0.009		
*LOC100038422*	0.025	1.28						
*Nup210l*	0.037	1.27						
*Kmo*	0.024	1.27						
*Tmprss11a*	0.049	1.26	11.83	0.01				
*BC094916*	0.036	1.26						
*Krt8*	0.013	1.25			3.12	0.008		
*Olfr539*	0.035	1.25						
*Slitrk6*	0.023	1.24			3.24	0.006		
*Cd209f*	0.031	1.24						
*Krt39*	0.008	1.23			3.05	0.009		
*Olfr121*	0.026	1.23						
*Gm11362*	0.041	1.23						
*Hcn4*	0.048	1.23			-2.95	0.007		
*Olfr1018*	0.022	1.23						
*Cdnf*	0.044	1.23						
*Casp3*	0.021	1.23						
*4933416I08Rik*	0.049	1.22						
*Vmn2r109*	0.022	1.22					1.14	0.008
*Stac*	0.029	1.22	20.18	0.007				
*Vmn1r5*	0.042	1.21						
*Dnm3os*	0.050	1.21					1.16	0.007
*Olfr648*	0.003	1.21						
*Olfr1131*	0.026	1.21						
*4930524N10Rik*	0.006	1.21						
*Gm4801*	0.011	1.21						
*Mrgprh*	0.007	1.21						
*Gm11437*	0.026	1.20						
*Apol7a*	0.010	1.20						
*C330022B21Rik*	0.020	-1.20						
*1600015I10Rik*	0.005	-1.20						
*Gm4776*	0.024	-1.20			-2.91	0.008		
*Olfr455*	0.011	-1.20						
*Olfr979*	0.007	-1.21						
*Mafg*	0.036	-1.21	9.39	0.009				
			-12.67	0.005				
			13.42	0.001				
*Olfr2*	0.022	-1.21	-10.92	0.001	3.21	0.006		
*Gm16551*	0.006	-1.22						
*4930401B11Rik*	0.047	-1.22						
*L3mbtl4*	0.040	-1.22	14.80	0.010				
*D4Wsu53e*	0.005	-1.22						
*Olfr281*	0.013	-1.24						
*D730002M21Rik*	0.045	-1.25						
*BC055004*	0.039	-1.25						
*Hdx*	0.015	-1.25						
*Olfr1350*	0.002	-1.26						
*Crygb*	0.011	-1.27						
*Tmem79*	0.027	-1.29						
*Zfa*	0.023	-1.31						
*Dnahc7a*	0.023	-1.39						

All identified differentially expressed genes are shown (fold-change cut off>1.2, *p*<0.05). Differentially 5-methylcytosine (5mC) methylated regions (DMRs) and regions of differentially histone modification (RDHMs) in gene promoters are also shown (cut-off *p*<0.01). Positive AMS indicates increased methylation in ethanol exposed mice, while positive MAT score indicates reduced methylation in ethanol exposed mice.

**Table 2:** MicroRNAs predicted to target mRNAs with reciprocal expression changes.

Gene expression			miRNA expression		
Gene Symbol	*p*-value	Fold change	miRNA ID	*p*-value	Fold change
*Hcn4*	0.048	1.23	*miR-185-5p*	0.026	-1.26
*Mafg*	0.036	-1.21	*miR-130a-3p*	0.018	2.19
			*miR-200b-3p*	0.022	1.95
*L3mbtl4*	0.040	-1.22	*miR-377-3p*	0.019	1.29
*Tmem79*	0.027	-1.29	*miR-34a-5p*	0.046	1.20

Genes and microRNAs (miRNAs) with reciprocal expression changes from each microarray experiment predicted to target genes are shown (fold-change cut off >1.2, p<0.05)

**Fig 2:**
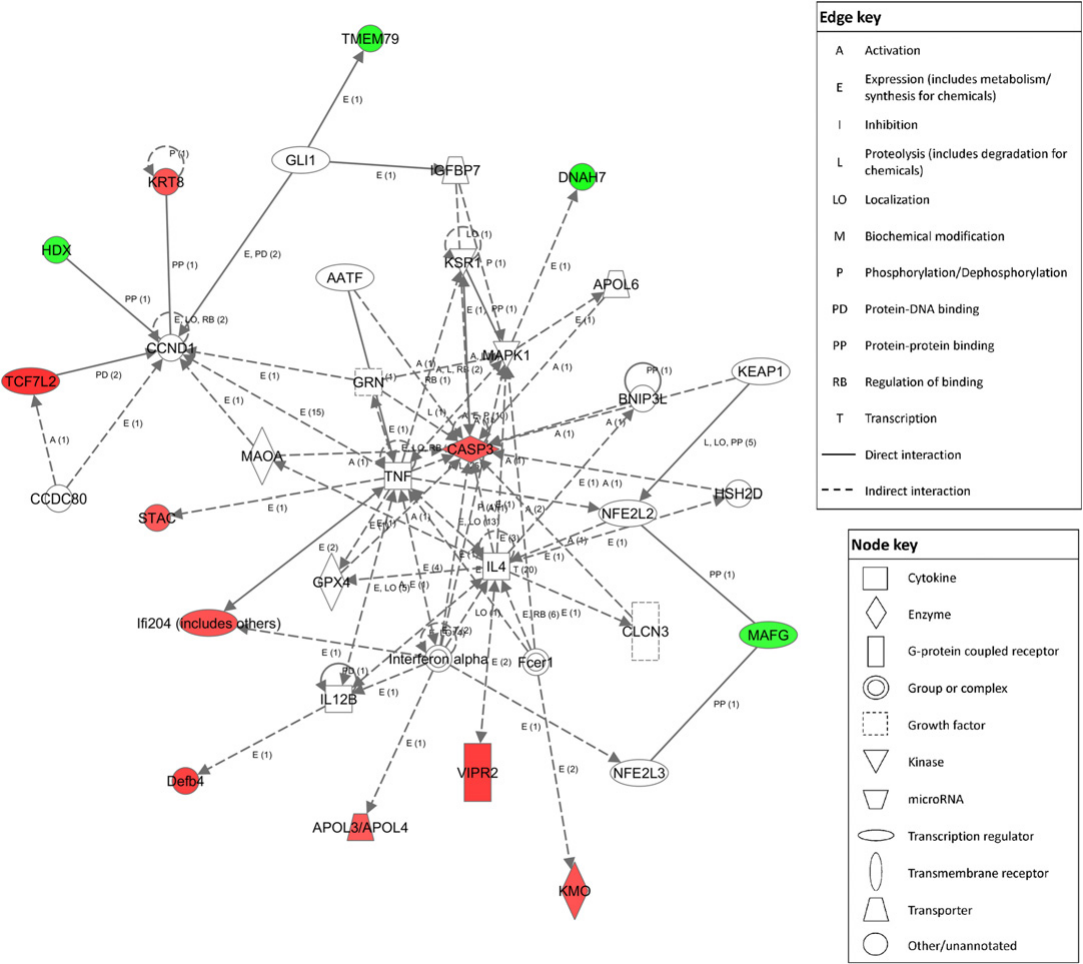
Top IPA network for gene expression changes “Free Radical Scavenging, Gene expression, Dermatological Diseases and Conditions”. Red nodes represent proteins whose transcripts were increased in ethanol-exposed mice vs. controls, green nodes represent those that were decreased in ethanol exposed mice. Score determined in IPA was 31 (right-tailed Fisher’s Exact Test).

The top enriched Partek pathways for the differentially expressed genes were “Olfactory Transduction”, “Colorectal Cancer”, and “Amoebiasis” (Table 3). The top enriched IPA network was “Free Radical Scavenging, Gene Expression, Dermatological Diseases and Conditions” (Fig 2). The top GO biological processes were determined in Enrichr (S1 Table). These processes include various metabolic and developmental pathways, of particular interest are apoptosis, oxidative stress response, and myelination. Top significant GO cellular components were also determined, all being classified as cell membrane or other structural components (S1 Table). Finally, the top GO molecular functions were determined, including various metabolite and protein binding domains, as well as membrane channels (S1 Table).

**Table 3:** Pathways significantly enriched with differentially expressed genes.

Pathway name	Number genes in list	Enrichment Score
**Partek pathway**		
Olfactory Transduction	10	14.2
Colorectal Cancer	2	4.4
Amoebiasis	2	3.8
**IPA**		
Free Radical Scavenging, Gene Expression, Dermatological Diseases and Conditions	12	31
Cellular Development, Developmental Disorder, Hereditary Disorder	2	3
Molecular Transport, RNA Trafficking, Cell Death and Survival	2	3
Cell Cycle, Nervous System Development and Function, Cell Signaling	2	3
Cardiovascular System Development and Function, Skeletal and Muscular System Development and Function, Cell-To-Cell Signaling and Interaction	2	2

Affected pathways and genes identified using both Partek and IPA network analysis software.

### DNA methylation analysis

MeDIP-chip and MEDME analysis identified a total of 4640 DMRs in gene promoters (defined as -5000 to 0 bp relative to the transcriptional start site) or gene bodies at AMS *p*-value<0.01. Of these DMRs, 82% were increases in methylation in ethanol-exposed mice. 549 DMRs also lay in annotated CpG islands, 93% of which were increases in methylation in ethanol-exposed mice at *p*<0.01. In addition, there were 126 DMRs in miRNA promoters/gene bodies, 65% of which were increases in methylation in ethanol-exposed mice. DMRs were nearly evenly distributed upstream and downstream of the TSS, with 48.9% of DMRs lying upstream. In addition, 23% of DMRs were within 1 kb of the TSS and 40% within 2 kb. The top 20 increased and decreased DMRs are shown (S2 Table).

We also assessed epigenetic changes using pathway analysis software as our past research indicates that it not only reflects possible transcriptional aberrations, but also contains an informative footprint of past transcriptional aberrations [29,31]. For pathway analysis, a more stringent AMS cut-off of *p*<0.001 was used. This DNA methylation pathway analysis list contained 697 genes. The top GO biological processes were determined in Enrichr, predominantly featuring growth, differentiation, and cell fate pathways (S3 Table). The top GO cellular components included membranes, and cell-to-cell connectivity proteins (S3 Table). GO molecular functions included hormone receptor binging, signalling protein binding, and transcription factors (S3 Table).

### Global H3K4 and H3K27 Methylation

For the histone methylation data, data were initially generated at a region with differential histone methylation (RDHM) *p-*value cut-off of *p*<0.01. For the H3K4me3 experiment, this level of significance identified 3398 unique RDHMs. 55% (1883) of these RDHMs had a negative model based analysis of tiling-array (MAT) score, indicating an increase in methylation in ethanol versus control mice. For the H3K27me3 experiment, 2268 unique RDHMs were identified and 11% (260) of RDHMs had a negative MAT score. 485 RDHMs overlap between the two methylations at *p*<0.01.

Next, we assessed the corresponding genes potentially affected by RDHMs. The H3K4me3 list contained 4092 unique genes, and the H3K27me3 experiment contained 2740 unique genes. There are more implicated genes than RDHMs because RDHMs are often in multiple gene promoters. For H3K4me3, 61% of RDHMs lied in gene promoters, 39% lied in gene bodies (including introns). For H3K27me3, 68% of RDHMs lied in gene promoters, while 32% lied in gene bodies. For pathway analysis, we again used a higher stringency MAT score cut-off of *p*<0.001. These lists were composed of 798 H3K4me3 genes and 223 H3K27me3 genes (Fig 1). The top 20 RDHM increases and decreases are shown for H3K4me3 (S4 Table) and H3K27me3 (S5 Table). The top GO functions for the H3K4me3 were heavily weighted towards synaptic structure and function and cell adhesion (S6 Table). This was due in large part to the presence of 13 protocadherin genes in the list. The H3K27me3 GO functions were also heavily cell-to-cell connectivity and synaptic in nature (S7 Table). This was due in part the presence of the same 13 protocadherin genes.

### Integrated Epigenetic Systems Analysis

**Fig 3:**
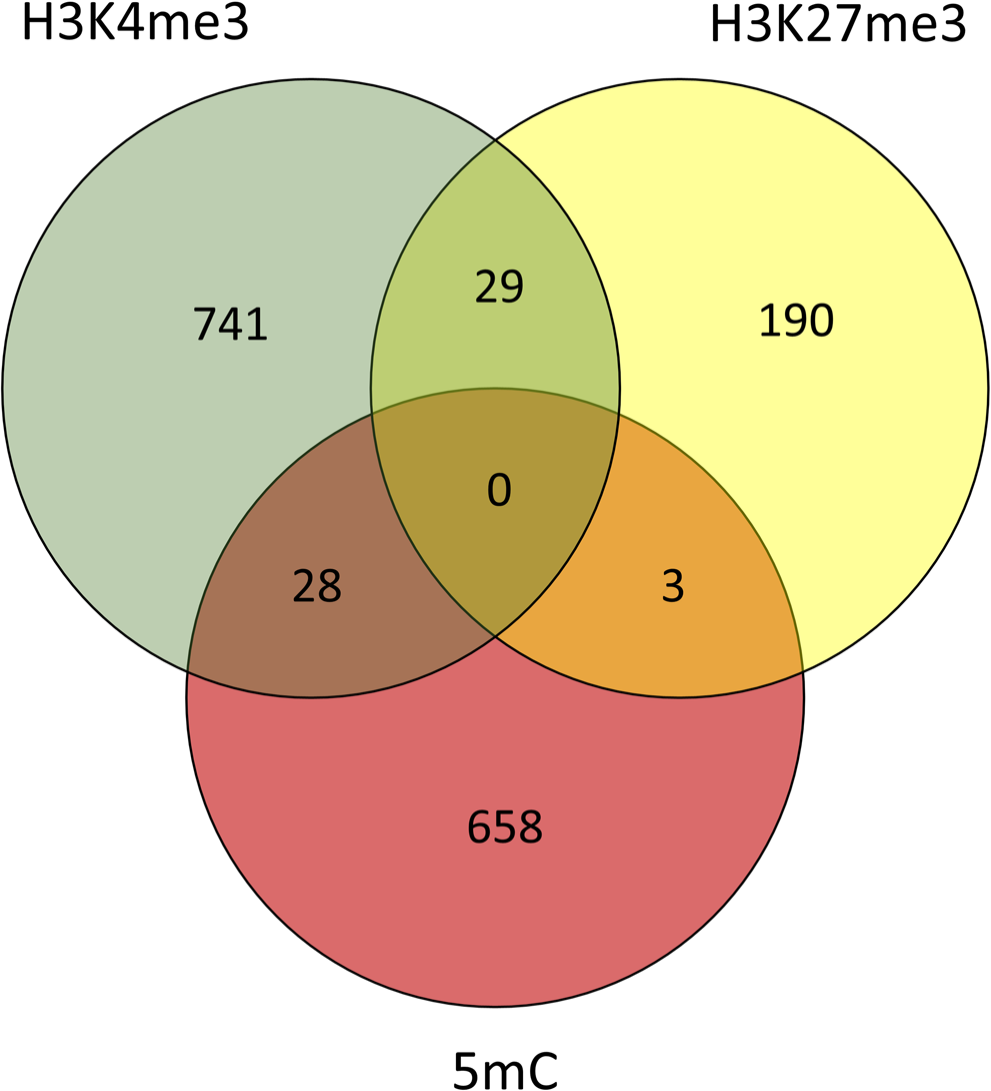
Combined gene list characterization, genes with either a DNA methylation, H3K4me3, or H3K27me3 change in their promoter. The number of genes proximal to each methylation change are shown in each circle. Genes proximal to multiple changes, regardless of the direction of those changes, are shown in overlapping regions.

**Fig 4:**
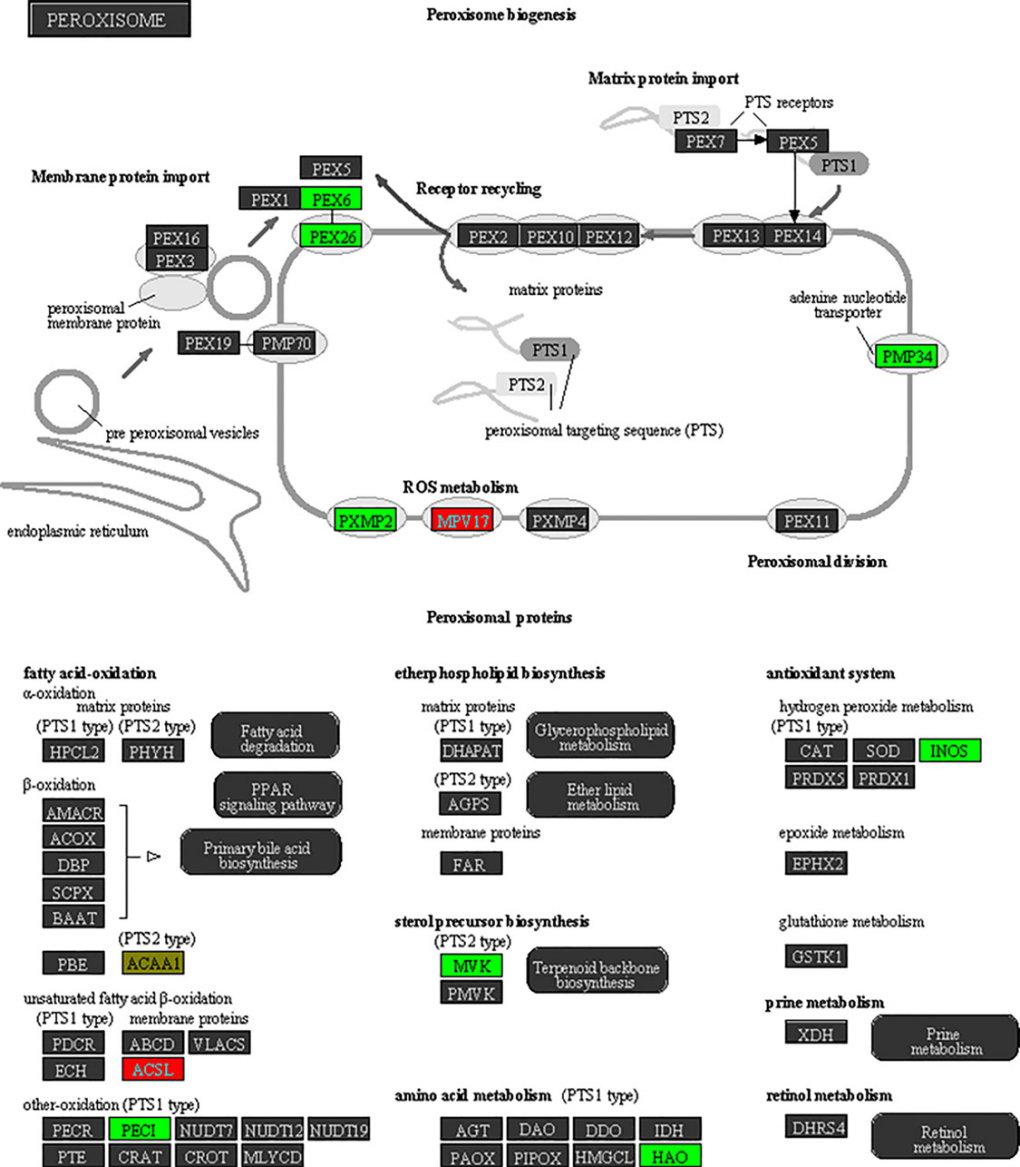
Schematic of peroxisome Biogenesis pathway from Partek pathway. Proteins are arranged into functional groups. Proteins whose genes bear DMRs or RDHMs in their promoter are colored: genes with a change predicted to increase gene expression are shown in red, those predicted to decrease are shown in green, conflicting marks are shown in yellow. Score determined in Partek was 5.4 (right-tailed Fisher’s exact test).

Since epigenetic marks act in concert, and do not exist in isolation, we assessed the changes in DNA methylation, H3K4me4, and H3K27me3 together [32]. To do this, we created a combined gene list of genes with either a DMR or an RDHM in their promoter/gene body. The direction of each change in ethanol-exposed mice was standardised between the marks by listing genes with changes predicted to increase gene expression as +1 (i.e. loss of DNA methylation, loss of H3K27me3, gain of H3K4me3) and changes predicted to decrease gene expression as -1 (i.e. gain of DNA methylation, gain of H3K27me3, loss of H3K4me3). Conflicting gains/losses were scored as 0 (22 genes total). The DMR/RDHM *p*-value cut off was kept at *p<*0.001. The list comprised 1589 genes (Fig 3). The top Partek pathway was Peroxisome (Fig 4), the top IPA network was Connective Tissue Disorders, Protein Synthesis, Cardiovascular System Development and Function (Table 4). The top 10 GO biological processes were determined in Enrichr, the top 4 of which are cell adhesion-related (S8 Table). Of note, there are also many neuron development pathways identified. The top GO cellular components were also determined, including many cellular support networks, and synaptic networks (S8 Table). Finally, the top 20 GO molecular functions were determined, including calcium ion binding, calmodulin binding, as well as various growth factor functions.

**Table 4:** Pathways significantly enriched with DMR- or RDHM-proximal genes.

Pathway name	Number genes in list	Enrichment Score
**Partek Pathway**		
Peroxisome	14	5.4
Hematopoietic cell lineage	13	4.6
Notch signalling pathway	8	3.7
ABC transporters	8	3.6
Jak-STAT signaling pathway	15	2.8
**IPA**		
Connective Tissue Disorders, Protein Synthesis, Cardiovascular System Development and Function	64	64
Cardiac Hypertrophy, Cardiovascular Disease, Developmental Disorder	60	56
Humoral Immune Response, Protein Synthesis, Hematological System Development and Function	56	49
Cellular Development, Cellular Growth and Proliferation, Hematological System Development and Function	51	41
Skeletal and Muscular Disorders, Developmental Disorder, Hereditary Disorder	43	30
Hematological System Development and Function, Tissue Morphology, Cell-To-Cell Signaling and Interaction	40	26
Endocrine System Development and Function, Molecular Transport, Protein Synthesis	38	24
Cell Death and Survival, Antimicrobial Response, Inflammatory Response	37	23
Cell-To-Cell Signaling and Interaction, Hematological System Development and Function, Immune Cell Trafficking	36	21
Embryonic Development, Organismal Development, Cell-To-Cell Signaling and Interaction	35	20
Cell-To-Cell Signaling and Interaction, Reproductive System Development and Function, Tissue Development	35	20
Cell Death and Survival, Lipid Metabolism, Small Molecule Biochemistry	35	20
Cell Cycle, DNA Replication, Recombination, and Repair, Cellular Development	34	19
Embryonic Development, Organismal Development, Cell Morphology	34	19
Cell Death and Survival, Cancer, Cellular Development	34	19
Cell-To-Cell Signaling and Interaction, Nervous System Development and Function, Behavior	33	18
Lipid Metabolism, Small Molecule Biochemistry, Molecular Transport	33	18
Cell Morphology, Cell Death and Survival, Nervous System Development and Function	31	16
Cell-To-Cell Signaling and Interaction, Nervous System Development and Function, Cellular Development	31	16
Lipid Metabolism, Small Molecule Biochemistry, Vitamin and Mineral Metabolism	30	15
Tissue Morphology, Embryonic Development, Organismal Development	30	15
Nervous System Development and Function, Cellular Development, Tissue Morphology	29	14
Cell Morphology, Cellular Compromise, Cellular Development	23	10

Affected pathways and genes in combined methylation gene list using both Partek and IPA analysis software.

### Gene-specific confirmations

**Fig 5:**
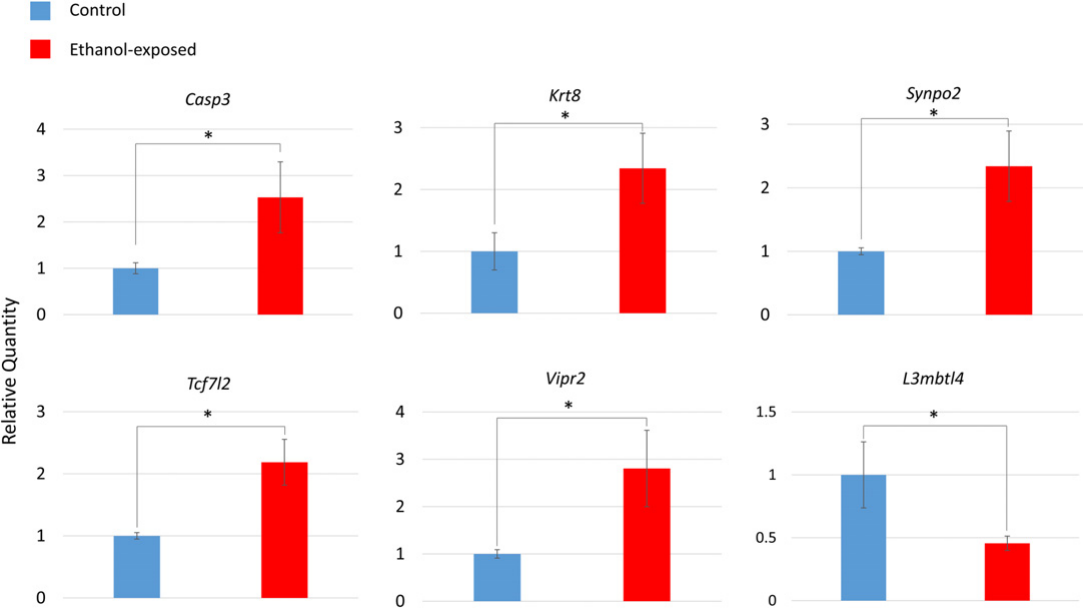
Droplet digital PCR (ddPCR) confirmation of differential gene expression. Data are normalized to a gene of interest relative quantity of 1.00 for the control group. n = 14, 7 ethanol-exposed and 7 control mice. Data are mean ± standard error. *p<0.05 (Student’s t-test).

We selected a subset of 10 gene expression changes to confirm using the same samples from the microarray by droplet digital PCR (ddPCR). Eight of these genes were selected from the top IPA network (Free Radical Scavenging, Gene Expression, Dermatological Diseases and Conditions) and two additional genes from the affected gene list. Up-regulation of 4 genes from the top IPA network were confirmed by ddPCR: *Casp3*, *Krt8*, *Tcf7l2*, and *Vipr2* (Fig 5). Each of the confirmed fold changes was greater than that indicated by the microarray (Table 1). In addition, up-regulation of *Synpo2* and downregulation of *L3mbtl4* were also confirmed (Fig 5). Four other gene expression changes were not confirmed; *Mafg*, *Tmem79*, and *Stac* were not significant, while *Defb4* transcript was not detected. The concentrations for each gene cDNA and the reference gene cDNA as calculated by the ddPCR system are also presented (S9 Table).

Five RDHMs were present in the 13 protocadherin genes implicated in the histone methylation data. We attempted to confirm the RDHM that overlapped with all 13 protocadherin genes, responsible for implicating sevens genes. This particular region was decreased in both H3K4me3 and H3K27me3 in ethanol-exposed mice. We designed primers to target the region with ChIP-qPCR, but the efficiency as measured by the standard curve was outside acceptable parameters.

**Fig 6:**
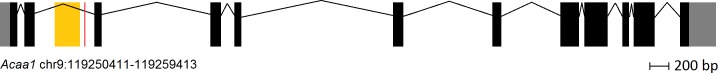
Location of differentially methylated CpG position in of *Acaa1* gene. Bars denote *Acaa1* exons, lines denote introns, grey bars denote untranslated regions, and black bars denote coding sequence. Yellow bar shows location of DMR from microarray. Red line shows location of 3.2% decrease in methylation at cytosine in CpG site in ethanol-exposed mice (Student’s t-test). Not pictured an additional DMR 3.7 kb upstream, 1.2 kb in size.

To confirm changes in DNA methylation at the nucleotide level, pyrosequencing of six specific MeDIP-chip DMRs was performed using the same samples from the microarray. We investigated CpGs in, and just outside the DMRs for three genes from the peroxisome pathway (*Pxmp2*, *Acaa1a*, and *Pex6*) and two genes from the top gene expression pathway (Free Radical Scavenging, Gene Expression, Dermatological Diseases and Conditions; *Tcf7l2* and *Mafg*). There was a significant (*p*<0.05, Student’s t-test) 3.2% decrease in the methylation of one CpG 30 nucleotides downstream of the implicated *Acaa1a* DMR in ethanol-exposed mice (position chr9:119342378). This CpG lies 959 bp downstream of the TSS of *Acaa1* (Fig 6). We also found a nominally significant (*p* = 0.057) 2.1% decrease in methylation at one cytosine (chr17:46706661) in the *Pex6* DMR. The mixing controls for these two SNPs showed the assay was highly predictive of methylation percentage (S10 Table).

## Discussion

We found hundreds of DNA methylation changes in gene promoters using MEDIP-chip. Interestingly, the changes were predominantly increases in methylation. This finding became more pronounced as the *p*-value cut-off of the DMR was increased (83% increased, *p*<0.001; Fig 1), and also remained true regardless of the region: gene promoters, CpG islands, and miRNA promoters. Many FASD methylation studies find global hypomethylation after ethanol exposure, consistent with ethanol-impaired cellular methylation processes. Our findings corroborate one of the few studies of similar design, which found hypermethylation in the hippocampus following neonatal ethanol exposure in a rat model of FASD [17]. The effect of ethanol on the methylome is not simple, with timing, dosage, and tissue/cell type offering dramatically different results. However, the findings may be reproducible with similar experimental designs. This hypermethylation may be explained be ethanol-induced changes in oxidative stress pathways, which also impact methyl donner metabolism [33]. It may be that this particular ethanol-exposure regime results in specific cellular conditions leading to DNA hypermethylation.

We also found hundreds of H3K4me3 and H3K27me3 changes in gene promoters using ChIP-chip. The majority of H3K4me3 changes (71% at *p*<0.001) were increases in methylation in ethanol-exposed mice (Fig 1). In contrast, the majority of H3K27me3 changes (92% at *p*<0.001) were decreases in methylation in ethanol-exposed mice (Fig 1). We performed GO analysis on each of the H3K4me3 and H3K27me3 gene lists individually. We found a great deal of cell-to-cell connectivity and synaptic functions. This was due in part to 13 protocadherin genes in both gene lists. Protocadherin (*Pcdh*) genes are believed to be responsible for establishing specific connections between neurons in vertebrate brain development by generating single-neuron diversity [34,35]. Since we were not able to confirm differentially methylation of *Pcdh* genes, were sought to confirm other genes relevant at the synapse. We confirmed upregulation of *Synpo2* (synaptopodin 2) in ethanol-exposed mice (Fig 5). Synaptopodins are a class of proteins that are highly expressed in telencephalic dendrites. The precise function of synaptopodins is unknown; they found at dendritic spines and post-synaptic densities [36,37]. *Synpo2* dysregulation may underlie some of their characteristic learning and memory impairment in P4,7 ethanol-exposed mice [38].

Ingenuity pathway analysis (IPA) identified the top affected gene expression network as “Free Radical Scavenging, Gene Expression, Dermatological Diseases and Conditions” (Fig 2). This gene network is responsible for coordinating the transcriptional free radical scavenging response. NFE2L2 homodimers and NFE2L2/MAFG heterodimers control the expression of genes with antioxidant response elements (ARE) in their promoters [39]. Such genes are involved in response to inflammation resulting from elevated free radical levels. Other proteins in this network have roles in oxidative stress such as GPX, KEAP1, and apolipoproteins. This network also includes many apoptosis-related proteins including BNIP3L, AATF, and HSD2D as well as genes important in the brain such as MAOA, CLCN3. Dysregulation of this pathway could impact these critical processes, all of which are relevant to FASD etiology.

Microarray analysis identified thirteen genes which were differentially regulated in this top IPA network. Four of these changes were confirmed by ddPCR: *Casp3*, *Krt8*, *Tcf7l2* and *Vipr2* (Fig 5); *Casp3* (Caspase-3) is a hub of this network. Caspase-3 has a key role in the execution phase of cellular apoptosis. Caspase-3 is inducible by oxidative stress [40], and its activation by ethanol is part of the apoptotic cascade that happens in the fetal brain during development [41]. TCF7L2 regulates insulin secretion, acting as a transcription factor in the Wnt pathway. Wnt signalling is key in brain development and synaptogenesis as well as adult functions such as synaptic modeling and neuronal maintenance [42]. VIPR2 is a G-protein coupled receptor for a small neuropeptide, pituitary adenylate cyclase activating polypeptide (PACAP). PACAP acts as a hypothalamic hormone, a neurotransmitter and a neurotrophic factor [43]. *Vipr2* showed methylation differences in a recent ADHD study in children [44]. We also confirmed the downregulation of *L3mbtl4* which is a putative polycomb group (PcG) protein. These proteins maintain repressive chromatin states by modification of histone modifications.

The top network from the Partek combined methylation analysis was “Peroxisome biogenesis” (Fig 4). Peroxisomes are membrane bound organelles found in all eukaryotic cells. Their main functions are the β-oxidation of very-long-chain fatty acids (VLCFAs) and synthesis of ether lipids such as plasmalogens [45]. The β-oxidation genes *Acaa1a* (Acetyl-CoA Acyltransferase 1A) and *Peci* were differentially methylated. Importantly, peroxisomes are key to the redox balance of the cell; both generating and scavenging free radicals [45]. The ROS-generating Nitric Oxide Synthase, *Nos2*, gene was differentially methylated in this study. NOS2 is also involved in neurotransmission [46]. Peroxisome production in response to oxidative stress is regulated by the *Pex* genes, which assemble peroxisome structure and guide matrix proteins inside the organelle. We found the *Pex26* and *Pex6* genes to be differently methylated. PEX26 is a peroxisome biogenesis factor that anchors PEX1 and PEX6 to the peroxisomal membrane, and is likely required for protein import [47].

We investigated several CpGs using pyrosequencing in the DMRs identified in the Peroxisome pathway. We confirmed a 3.2% decrease in the methylation of one CpG in the *Acaa1* regulatory region. As stated above, this gene is critical for the peroxisomal β-oxidation. We did not identify any other significant changes, though one nominally significant (*p* = 0.057) decrease in methylation occurred at one cytosine in the *Pex6* DMR. One possible reason for not identifying more methylation changes by pyrosequencing was the use of anti-methylcytosine antibody for the MeDIP-chip. This would allow any differences in cytosine methylation, not just CpG cytosines, to be identified by MeDIP analysis. Non-CpG methylation is highly abundant in the brain, representing 25% of all cytosine methylation in hippocampal dentate granule neurons [48].

Oxidative stress is a well characterized component of FASD etiology. Ethanol acts directly on mitochondria to produce superoxide, hydroxide, and nitric oxide radicals [49]. Metabolism of ethanol by cytochrome P450 2E1 produces oxidized products and ultimately hydroxide radial generation [50]. Catalase also produces acetaldehyde from alcohol in the brain, further increasing the formation of ROS [51]. Oxidative damage can lead to blood-brain barrier impairment, inflammation, and increased apoptosis [52]. Interestingly, these are also key features of FASD etiology. Indeed, oxidative damage is observed in many rodent models of FASD, including lipid peroxidation, protein oxidation, and DNA damage [12]. Lipid peroxidation is not often present in young animals, but accumulates over time into adulthood [4]. In a *Drosophila* model of developmental ethanol exposure, changes in expression of antioxidant genes contributed to oxidative stress in adult flies [53]. Further, this increased oxidative stress was a primary cause of developmental delay associated with ethanol exposure [53]. Taken with our results, there is mounting evidence that ethanol causes lasting and functionally relevant oxidative damage to the brain dependent on gene expression changes.

**Fig 7:**
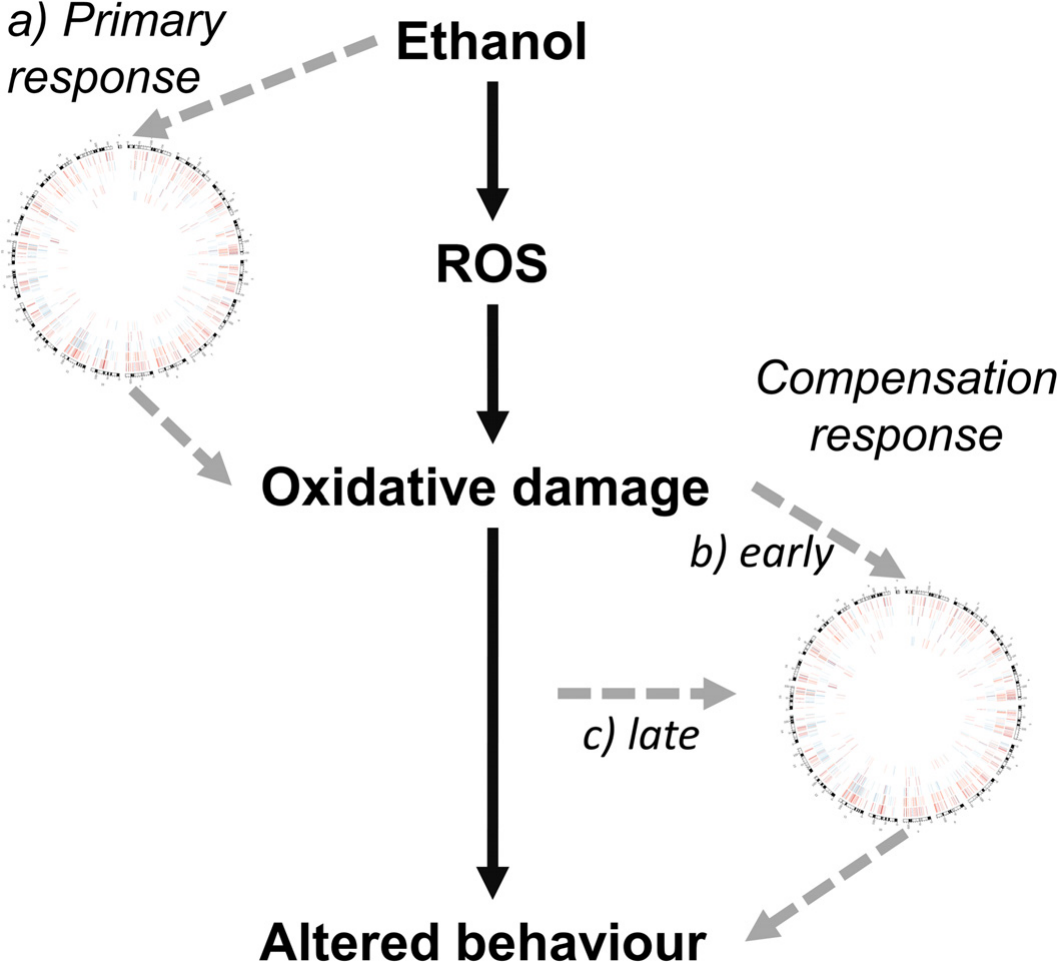
Potential origins of observed epigenetic and gene expression hippocampal profile in response to neonatal ethanol exposure. It is well established that in the brain ethanol leads to increased ROS, leading to oxidative damage, which contributes to altered behaviour. The epigenetic and gene expression changes identified here (represented by the Circos plot from Fig 1) may have arisen from: a) the direct action of ethanol during the exposure period, which may then act to perpetuate ethanol-induced oxidative damage; b) an early response to ethanol-induced oxidative cellular damage, acting to ameliorate or compensate for this damage; c) an later response to accumulating oxidative damage over the early life of the mouse, prior to 70 days of age.

There are three main possibilities for the origin of these expression and epigenetic changes (Fig 7). First, these changes were established as a direct response ethanol during exposure and are maintained to adulthood. Second, these changes were indirectly caused by ethanol as a compensation or amelioration response to ethanol-induced oxidative stress. Third, these changes presented later in life in response to long-term accumulation of oxidative damage. We believe that the second explanation is most likely, or perhaps a combination of the three. As previously discussed, ethanol is known to induce ROS as one of its primary effects on the brain. The genes involved in the response to this stress include those differentially methylated/expressed in this study. Cells may have altered the epigenetic regulation of these genes to cope with oxidative stress and its effects. Future experiments should be designed to distinguish between these possible explanations, such as investigating several time points after ethanol exposure.

The data presented here show that fetal alcohol exposure has a lasting impact on the hippocampal transcriptome and epigenome. While few individual genes were identified across the expression, DNA methylation, and histone methylation analyses, highly similar biological processes were affected. We report a novel interface of free-radical scavenging and epigenetic mechanisms, two key processes in FASD etiology. The implication of differentially regulated free-radical scavenging pathways suggests an altered free-radical scavenging response lasting into adulthood. Ultimately, a better understanding of the dynamics of these relationships could lead to novel biomarkers or therapeutic targets, neither of which have been developed for FASD. More broadly, the data provide a better understanding of the complex responses of the epigenome to the environment.

## Materials and Methods

### Mouse care

Protocols were approved by the Animal Use Subcommittee (AUS) at the University of Western Ontario, London, Ontario, Canada. C57BL/6J (B6) mice were originally obtained from Jackson Laboratories (Bar Harbor, MA) and a population was subsequently maintained at the Animal Care Facility at the University of Western Ontario. Female mice age 12–18 weeks were separated into individual standard shoebox housing and mated with males of approximately the same age. The day of birth was termed post-natal day (PD) zero.

Sex and weight-matched littermate pups were divided into two groups: ethanol-treated and saline control. Pups were given two subcutaneous dorsal injections at 9 am and 11 am on both PD4 and PD7. Ethanol-treated mice were injected with 2.5 g/kg of ethanol in 0.15 M NaCl [54]. This protocol produces blood alcohol concentrations above the toxic threshold of 200 mg/dl for over eight hours [23]. Control mice were injected with 0.15 M saline. Pups were weaned on PD21 and housed in cages of two to four same-sex littermates. Male mice were used for all subsequent analyses (n = 18). Mice were sacrificed on PD 70 via carbon dioxide asphyxiation. This time point was chosen because it is the onset of adulthood, and indicates that changes have been maintained through adolescent development. The hippocampus was dissected out [55], snap-frozen in liquid nitrogen, and stored at -80°C.

### DNA/RNA isolation

DNA and RNA were isolated with AllPrep DNA/RNA Mini Kit (Qiagen, Valencia, CA, USA) according to the manufacturer’s protocol. This kit allows DNA and RNA to be isolated from the same hippocampal sample. DNA and RNA were stored at -20°C and -80°C respectively.

### Gene and miRNA expression microarray

Nine ethanol-exposed and nine control hippocampus samples were used for expression analysis. RNA quality was assessed using the Agilent 2100 Bioanalyzer (Agilent Technologies Inc., Palo Alto, CA) and the RNA 6000 Nano kit (Caliper Life Sciences, Mountain View, CA). RNA from three non-littermate males was then pooled for microarray analysis on three separate arrays per treatment group.

All sample labeling and GeneChip processing was performed at the London Regional Genomics Centre (Robarts Research Institute, London, Ontario, Canada; http://www.lrgc.ca). RNA quality was assessed using the Agilent 2100 Bioanalyzer (Agilent Technologies Inc., Palo Alto, CA) and the RNA 6000 Nano kit (Caliper Life Sciences, Mountain View, CA). Single stranded complimentary DNA (sscDNA) was prepared from 200 ng of total RNA as per the Ambion WT Expression Kit for Affymetrix GeneChip Whole Transcript WT Expression Arrays (http://www.ambion.com/techlib/prot/fm_4411973.pdf, Applied Biosystems, Carlsbad, CA) and the Affymetrix GeneChip WT Terminal Labeling kit and Hybridization User Manual (http://media.affymetrix.com/support/downloads/manuals/wt_term_label_ambion_user_manual.pdf, Affymetrix, Santa Clara, CA).

Total RNA was first converted to cDNA, followed by *in vitro* transcription to make cRNA. 5.5 μg of single stranded cDNA was synthesized, end labeled and hybridized, for 16 hours at 45°C, to Mouse Gene 1.0 ST arrays. One microgram of total RNA was labeled using the Flash Tag Biotin HSR kit from Genisphere (http://www.genisphere.com/array_detection_flashtag_biotin.html). Samples were then hybridized to Affymetrix miRNA 2.0 arrays for 16 hours at 48°C. All washing steps were performed by a GeneChip Fluidics Station 450 and GeneChips were scanned with the GeneChip Scanner 3000 7G (Affymetrix, Santa Clara, CA) using Command Console v1.1.

Probe level (.CEL file) data was generated using Affymetrix Command Console v1.1. Probes were summarized at the miRNA and gene level using RMA [56]. Partek was used to determine ANOVA *p*-values and fold changes for genes and miRNAs. Species annotations were added and used to filter miRNAs. Partek Pathway was used to determine and visualize significantly enriched pathways (using a Fisher’s exact test). CEL files and log2 normalized files were uploaded to GEO.

### Droplet-Digital PCR

Purified RNA was converted to cDNA using the High-Capacity cDNA Reverse Transcription Kit (Thermo-Fisher). cDNA was diluted 10-fold and stored at -20°C until use. Individual genes were investigated with TaqMan® assays (Applied Biosystems), assays IDs: *Vipr2*: Mm01238618_g1; *Synpo2*: Mm03809162_m1; *Tcf7l2*: Mm00501505_m1; *Casp3*: Mm01195085_m1, *Krt8*: Mm04209403_g1; *L3mbtl4*: Mm00623914_m1, *Stac*; Mm00450338_m1, *Mafg*: Mm00521961_g1, *Tmem79*: Mm00470361_m1, *Defb4*: Mm00731768_m1. For all assays, Tata Binding Protein (TBP) was used as a reference gene: Mm01277042_m1.

For each assay, the gene of interest and TBP reference gene were run in multiplex using FAM and VIC labeling respectively. Reactions were prepared using ddPCR™ Supermix for Probes (BioRad), DNA, and probes according to the manufactures protocol. Droplets were generated from the reactions using Droplet Generation Oil for Probes (BioRad) on the QX100 Droplet Generator (BioRad) according to the manufacture’s protocol. Droplets were cycled on the C1000 Touch Thermal Cycler (BioRad) for 40 cycles, 60°C annealing temperature, 2°C/sec ramp speed. Droplets were read using the QX100 Droplet Reader (BioRad). Data were analyzed in QuantaSoft software (BioRad). All samples had between 17000–20000 droplets indicating high-quality. The concertation of each RNA species and ratio of gene of interest/reference gene concentration was calculated using QuantaSoft for each sample. Each DNA sample was run in three technical replicates, the average ratio across technical replicates for each sample was calculated manually. Each DNA sample’s average ratio was used to compare ethanol-exposed (n = 7) to control (n = 7) samples using a Student’s t-test. Averages were normalized to 1.00 relative expression level for control group.

### MeDIP-chip

#### Genomic DNA Fragmentation

Genomic DNA (gDNA) was quantified and quality assessed by NanoDrop ND-1000. Genomic DNA of each sample was sonicated to ~200–1000 bp with a Bioruptor sonicator (Diagenode) on “Low” mode for 10 cycles of 30 seconds “ON” & 30 seconds “OFF”. The gDNA and each sheared DNA were agarose analyzed.

#### Methyl-cytosine Immunoprecipitation

1 μg of sonicated genomic DNA was used for immunoprecipitation using a mouse monoclonal anti-5-methylcytosine antibody (Diagenode). For this, DNA was heat-denatured at 94°C for 10 min, rapidly cooled on ice, and immunoprecipitated with 1 μL primary antibody overnight at 4°C with rocking agitation in 400 μL immunoprecipitation buffer (0.5% BSA in PBS). To recover the immunoprecipitated DNA fragments, 200 μL of anti-mouse IgG magnetic beads were added and incubated for an additional 2 hours at 4°C with agitation. After immunoprecipitation, a total of five immunoprecipitation washes were performed with ice-cold immunoprecipitation buffer. Washed beads were resuspended in TE buffer with 0.25% SDS and 0.25mg/mL proteinase K for 2 hours at 65°C and then allowed to cool down to room temperature. MeDIP DNA were purified using Qiagen MinElute columns (Qiagen).

#### Whole Genome Amplification (WGA)

The MeDIP-enriched DNA was amplified using a WGA kit from Sigma-Aldrich (GenomePlex® Complete Whole Genome Amplification (WGA2) kit) following manufacturer’s protocol. The amplified DNA samples were then purified with QIAquick PCR purification kit (Qiagen) following manufacture’s protocol.

#### Real-time PCR assessment of fold-enrichment

The purpose of the qPCR experiment is to verify the MeDIP DNA has been enriched for methylated fragments and depleted for unmethylated fragments [57]. The primers for specifically methylated region (the positive control, *Tsh2b* promoter) and unmethylated region (the negative control, *Gapdh* promoter) are used to assess the enrichment level of these two regions in both Input (sonicated DNA) and MeDIP enriched DNA [57]. All six samples showed expected enrichment (S11 Table). An enrichment value for two samples could not be calculated due to complete lack of amplification in the IgG negative control. All samples can be considered quantitatively above the background signal (noise) for each. The PCR primer sequences were: *Tsh2b* 101bp F:5’CTCTCCTTGCGGCATCTCT3’ R:5’GCGGTAAAGGGTGCTACTATT3’. *Gapdh* 161bp F:5’GCCCTTGAGCTAGGACTGGATAA3’ R:5’CCTGGCACTGCACAAGAAGATG3’.

#### DNA Labelling and Array Hybridization

The purified DNA was quantified using a NanoDrop ND-1000. For DNA labelling, the NimbleGen Dual-Color DNA Labeling Kit was used according to the manufacturer’s guideline detailed in the NimbleGen MeDIP-chip protocol (NimbleGen Systems, Inc., Madison, WI, USA). 1 μg DNA of each sample was incubated for 10 min at 98°C with 1 OD of Cy5-9mer primer (IP sample) or Cy3-9mer primer (Input sample). Then, 100 pmol of deoxynucleoside triphosphates and 100U of the Klenow fragment (New England Biolabs, USA) were added and the mix incubated at 37°C for 2 hours. The reaction was stopped by adding 0.1 volume of 0.5 M EDTA, and the labeled DNA was purified by isopropanol / ethanol precipitation. Microarrays were hybridized at 42°C during 16 to 20h with Cy3/5 labelled DNA in NimbleGen hybridization buffer/ hybridization component A in a hybridization chamber (Hybridization System—NimbleGen Systems, Inc., Madison, WI, USA). Following hybridization, washing was performed using the NimbleGen Wash Buffer kit (NimbleGen Systems, Inc., Madison, WI, USA). For array hybridization, Roche NimbleGen's MM9 Meth 2.1M CpG plus Promoter array was used.

#### Data Extraction and Normalization

Raw data was extracted as pair files by NimbleScan software. We ArrayStar performed Median-centering, quantile normalization, and linear smoothing by Bioconductor packages Ringo, limma, and MEDME. After normalization, a normalized log2-ratio data (*_ratio.gff file) was created for each sample. From the normalized log2-ratio data, a sliding-window peak-finding algorithm provided by NimbleScan v2.5 (Roche-NimbleGen) was applied to find the enriched peaks with specified parameters (sliding window width: 750bp; mini probes per peak: 2; *p*-value minimum cut-off: 2; maximum spacing between nearby probes within peak: 500bp). Raw and normalized data files were uploaded to GEO.

#### MEDME analysis

To accurately quantify CpG methylation levels, we used MEDME (modeling experimental data with MeDIP enrichment) to improve the evaluation and interpretation of MeDIP derived DNA methylation estimates. MEDME relies on generating a fully methylated gDNA sample for comparison. To generate the fully methylated profiles, DNA from each sample was pooled and treated with CpG methyltransferase (M.SssI, NEB) to add methyl-groups to all cytosine residues within CpG di-nucleotides, in order to obtain fully methylated genomic DNA. Raw data for fully methylated sample and test samples were Median-centered and quantile normalized using Bioconductor packages Ringo and limma. Then MEDME was performed to calculate probe AMS and RMS. S1 Fig shows a logistic model to describe the association between MeDIP of log2R and the log2 observed methylation level using fully methylated genomic DNA experiment data. In the fully methylated DNA MeDIP experimental dataset, the weighted count of methylated CpG di-nucleotides in the 1 kb window centered at each probe is calculable by genomic CpG in the window, as every CpG is expected to be methylated (S1 Fig).

The MEDME utilizes the absolute methylation score (AMS) as the indicative of DNA methylation, which is decided by the weighted count of methylated CpG di-nucleotides in a 1 kb window centered at each probe. The AMS is verified to be a more accurate and sensitive indicative of DNA methylation than log-Ratio.

The MEDME method also provides a relative methylation score (RMS) that normalizes AMS with respect to the total number of CpGs represented by CpGw. Differentially methylated probes between ethanol-exposed and control groups were identified using AMS by t-test. And probes with *p*-value<0.05 and ABS (AMS_dif)>8 were selected and used to find AMS DMRs. The RMS is more useful when comparing regions with different CpG densities. Since we are only comparing the same region across samples, we use only AMS in our further characterization and analysis. MEDME array data was uploaded to GEO.

After probe AMS and RMS were obtained from analyzing the MeDIP-chip data by MEDME, a further analysis of identification of DMRs (differentially methylated region) was performed to identify significantly differentially methylated regions. We calculated two types of DMRs using AMS and RMS. Then DMRs are mapped to genomic features: transcripts, CpG islands and miRNAs.

### Sodium bisulfite pyrosequencing

The same DNA samples used for MeDIP-chip were used for sodium bisulfite pyrosequencing. EpigenDx performed pyro-sequencing on the PSQ96 HS System (Qiagen) following the manufacturer’s instructions, using custom assays and a gradient of controls with known methylation levels. This allowed for the quantification of the absolute percent methylation [58] of each CpG at specific loci using QCpG software (Qiagen). The absolute percent methylation at each assayed cytosine was averaged among ethanol-exposed (n = 3) and control (n = 3) samples and compared using a Student’s t-test. The custom primers assayed CpGs at the following positions (mm10): *Acaa1*: chr9:119342321, chr9:119342332, chr9:119342352, chr9:119342366, chr9:119342378, chr9:119342386; *Pxmp1*: 110285970, chr5110285964, chr5110285959, chr5110285948, chr5110285944, chr5110285940, chr5110285908, chr5110285878; *Pex6*: chr17:46706646, chr17:46706654, chr17:46706661, chr17:46706672, chr17:46706678, chr17:46706691, chr17:46706698, chr17:46706715; *Mafg*: chr11:120625270, chr11:120625264, chr11:120625261, chr11:120625225, chr11:120625205, chr11:120625131; *Tcf7l2*: chr19:55745017, chr19:55745023.

### ChIP-chip

#### Chromatin Immunoprecipitation

Hippocampal tissue samples were thawed on ice then treated with 1% formaldehyde for five minutes and sonicated with the truChIPTM Tissue Prep Kit for SDS Chromatin Shearing (Covaris) and the Covaris® S2 Sonicator (Woburn, MA, USA) according to the manufacturer’s protocol. The EpiQuik™ Tissue Chromatin Immunoprecipitation Kit (Epigentek) was used to perform ChIP. After sonication, samples were divided and immunoprecipitated with ChIP-grade polyclonal antibodies anti-H3K4me3 (Epigentek cat # A-4033) and anti-H3K27me3 (Millipore cat #07–499). Two microarray experiments were performed, one for each methylation state using the same chromatin sample from the same mice for each. Immunoprecipitated samples were sent to ArrayStar Inc. (Rockville, MD, USA). ArrayStar performed whole-genome amplification, target preparation DNA labelling and array hybridization.

#### Whole Genome Amplification (WGA)

The enriched DNA was amplified using a WGA kit from Sigma-Aldrich (GenomePlex® Complete Whole Genome Amplification (WGA2) kit). The amplified DNA samples were then purified with QIAquick PCR purification kit (Qiagen).

#### DNA Labelling

The NimbleGen Dual-Color DNA Labeling Kit was used according to the manufacturer’s NimbleGen ChIP-on-chip protocol (Nimblegen Systems, Inc., Madison, WI, USA). One μg of DNA from each sample was incubated for 10 min at 98°C with 1 OD of Cy5-9mer primer (IP sample) or Cy3-9mer primer (input sample). Then, 100 pmol of deoxynucleoside triphosphates and 100U of the Klenow fragment (New England Biolabs, USA) were added and incubated at 37°C for 2 hours. The reaction was stopped by adding 0.1 volume of 0.5 M EDTA. The labelled DNA was purified by isopropanol/ethanol precipitation.

#### ChIP microarray Hybridization

Microarrays were hybridized at 42°C for four hours with 4μg of Cy3/5 labelled DNA in Nimblegen hybridization buffer/ hybridization component A in a hybridization chamber (Nimblegen Systems, Inc., Madison, WI, USA). Washing was performed after hybridization using the Nimblegen Wash Buffer kit (Nimblegen Systems, Inc., Madison, WI, USA). For array hybridization, Roche NimbleGen's Mouse ChIP-chip 2.1M Deluxe Promoter Array was used. Samples were pooled in triplicate and hybridized to three arrays for each treatment; i.e. 9 ethanol-treated mice on three arrays were compared to 9 litter-matched controls on three arrays. Scanning was performed with the Axon GenePix 4000B microarray scanner. Raw data was extracted as pair files by NimbleScan software. The files were uploaded to GEO.

#### ChIP microarray analysis

The pair files were analyzed utilizing the tiling workflow provided in Partek Genomics Suite® version 6.6 (St. Louis, Missouri, USA). Nimblegen.pair files (representing the 635 nm and 532 nm scans) for each sample were normalized using the default methods of normalization in the tiling workflow in Partek. The default method includes adjustments for probe sequence, background correction, quantile normalization, and Log (base 2) transformation. In addition, to ensure quality, Principal Components Analysis (PCA) was performed. Files were annotated against mm9 and enriched regions were detected using a one-way ANOVA to compare enrichment between the ethanol-exposed and control groups: three ethanol-exposed mouse arrays contrasted to the three matched control mouse arrays. The enriched regions settings were set at a minimum *p*-value of 0.01 and the number of probes to call a region was set at a minimum of five. The Model-based Analysis of Tiling-arrays (MAT) algorithm was used to detect enriched regions [59]m. The MAT algorithm is designed to detect enriched regions in tiling ChIP-chip experiments, and provides a score for the degree of enrichment between experimental samples or groups of samples. A list of regions with MAT scores and corresponding p-values was output. These regions with differential histone methylation (RDHMs) were scored to overlap with RefSeq (2014-01-03 version) genes that when they occurred either within the gene body or 5000 bp upstream– 3000 bp downstream of the transcriptional start site. The list of gene names overlapping RDHMs with a MAT *p*-value<0.001 were generated.

The list of gene names from Partek were submitted as text files to Ingenuity Pathway Analysis (Ingenuity Systems Inc, CA, USA), Partek Pathway (Fishers Exact Test), and Enrichr [60] to determine overrepresented genes using gene ontology and other analyses. A cut-off of *p*<0.05 was used to determine significant pathways for all software programs.

## Supporting Information

S1 Fig The logistic model (blue line) describes the association between MeDIP log2R and the log2 observed methylation level. R is the log Ratio of MeDIP versus Input and mCG is the weighted count of methylated CpG in the 1 kb window centered at each probe.
(TIF)

S1 Table Gene ontology (GO) analysis of differentially expressed genes. Top 10 GO processes are shown where number of entries exceeds 10.
(DOCX)

S2 Table Top 20 increased and decreased differentially methylated regions (DMRs) from MeDIP-chip microarray analysis. The top and bottom 20 differentially methylated regions (DMRs) according to AMS are shown with the proximal gene including distance to the gene transcriptional start site (TSS).
(DOCX)

S3 Table Gene ontology (GO) analysis of genes with differentially methylated regions (DMRs) in their promoter. Top 10 GO processes are shown where number of entries exceeds 10.
(DOCX)

S4 Table Top 20 increased and decreases in H3K4me3 methylation from ChIP-chip microarray analysis. The top and bottom 20 regions of differential histone methylation (RDHMs) according to MAT score are shown with the proximal gene including distance to the gene transcriptional start site (TSS).
(DOCX)

S5 Table Top 20 increases and decreases in H3K27me3 methylation from ChIP-chip microarray analysis. The top and bottom 20 regions of differential histone methylation (RDHMs) according to MAT score are shown with the proximal gene including distance to the transcriptional start site (TSS).
(DOCX)

S6 Table Gene ontology (GO) analysis of genes with H3K4me3 RDHMs in their promoter. Top 10 GO processes are shown where number of entries exceeds 10.
(DOCX)

S7 Table Gene ontology (GO) analysis of genes with H3K27me3 RDHMs in their promoter. Top 10 GO processes are shown where number of entries exceeds 10.
(DOCX)

S8 Table Gene ontology (GO) analysis of genes with either a DMR or RDHM in their promoter. Top 10 GO processes are shown for each.
(DOCX)

S9 Table Absolute concentrations of mRNA species from droplet digital PCR (ddPCR). Concentrations for each gene of interest (GOI) and the reference gene Tata-Binding Protein (TBP) are shown for each experiment. The mRNA concentration is presented as an average of the concentration of each of seven replicates in each group. The standard error of the mean (SEM) is also presented. Each replicate was also calculated as an average of three separate technical replicates.
(DOCX)

S10 Table Pyrosequencing mixing control for cytosines of interest. Pyrosequencing was performed on DNA mixing controls of known methylation percentage were sequenced for each SNP. The r^2^ coefficient for each SNP is shown.
(DOCX)

S11 Table Real-time PCR assessment of MeDIP fold enrichment. Threshold cycle (Ct) values for each primer pair for each sample are shown.
(DOCX)
