# Supplementary figures and images for: Alteration of Gene Expression, DNA Methylation, and Histone Methylation in Free Radical Scavenging Networks in Adult Mouse Hippocampus following Fetal Alcohol Exposure
Source: PLoS One. 2016 May 2;11(5):e0154836. doi: 10.1371/journal.pone.0154836 (PMC4852908; doi:10.1371/journal.pone.0154836)

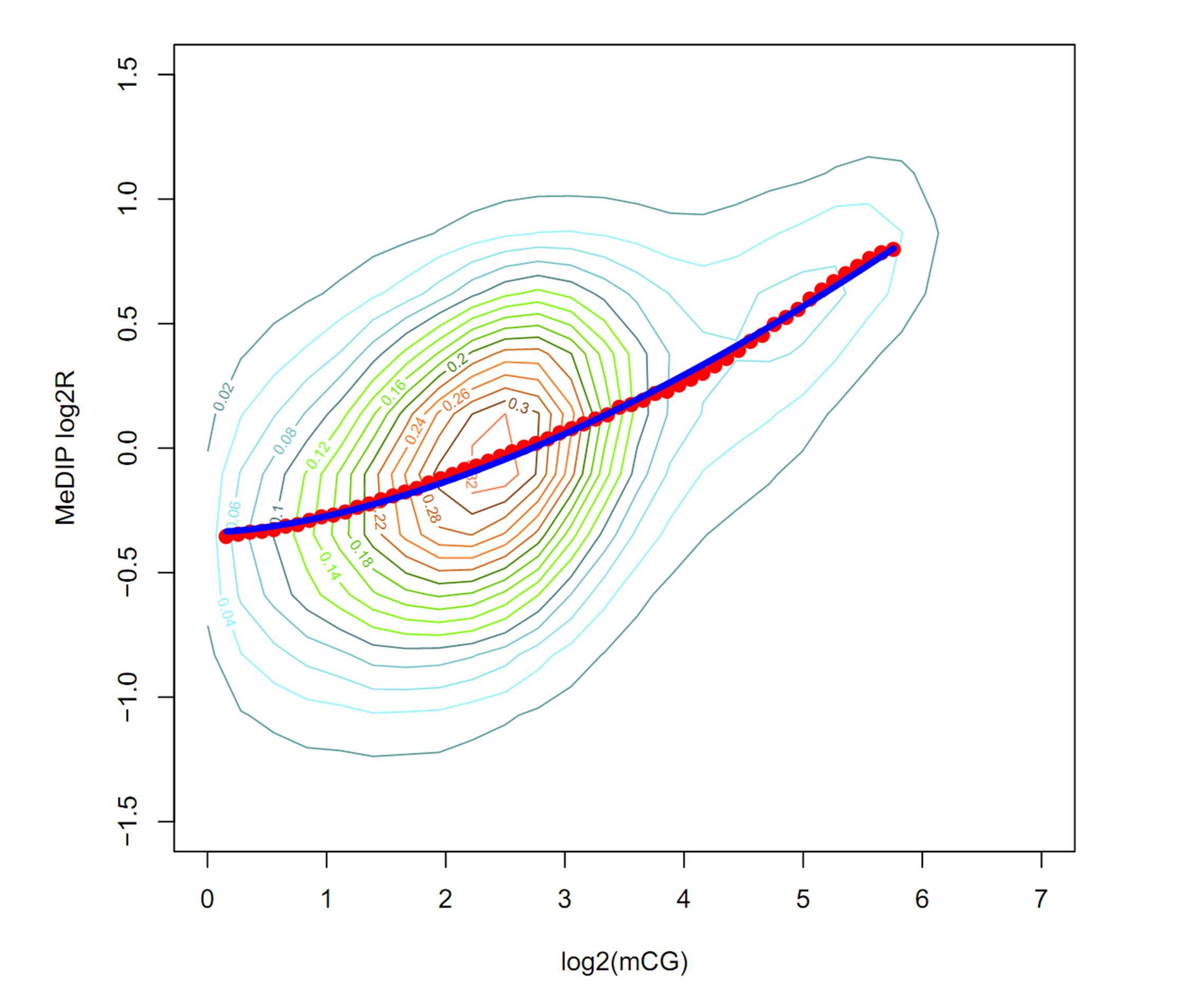

Supplement: S1 Fig — R is the log Ratio of MeDIP versus Input and mCG is the weighted count of methylated CpG in the 1 kb window centered at each probe. (TIF) [file pone.0154836.s001.tif]
