# Supplementary material for: Alteration of Gene Expression, DNA Methylation, and Histone Methylation in Free Radical Scavenging Networks in Adult Mouse Hippocampus following Fetal Alcohol Exposure
Source: PLoS One. 2016 May 2;11(5):e0154836. doi: 10.1371/journal.pone.0154836 (PMC4852908; doi:10.1371/journal.pone.0154836)
Supplement: S1 Table — Top 10 GO processes are shown where number of entries exceeds 10. (DOCX) [file pone.0154836.s002.docx]

**Table S1. Gene ontology (GO) analysis of differentially expressed genes.**

| **GO term** | **Process** | ***p-*value** |
| --- | --- | --- |
| Z disc (GO:0030018) | *GO cellular component* | 0.004 |
| intracellular cAMP activated cation channel activity (GO:0005222) | *GO molecular functions* | 0.008 |
| muscle alpha-actinin binding (GO:0051371) | *GO molecular functions* | 0.009 |
| armadillo repeat domain binding (GO:0070016) | *GO molecular functions* | 0.009 |
| cyclic nucleotide-gated ion channel activity (GO:0043855) | *GO molecular functions* | 0.010 |
| intracellular cyclic nucleotide activated cation channel activity (GO:0005221) | *GO molecular functions* | 0.010 |
| dicarboxylic acid biosynthetic process (GO:0043650) | *GO biological processes* | 0.011 |
| genitalia morphogenesis (GO:0035112) | *GO biological processes* | 0.011 |
| gamma-catenin binding (GO:0045295) | *GO molecular functions* | 0.011 |
| cyclin-dependent protein serine/threonine kinase inhibitor activity (GO:0004861) | *GO molecular functions* | 0.011 |
| positive regulation of gluconeogenesis (GO:0045722) | *GO biological processes* | 0.013 |
| response to auditory stimulus (GO:0010996) | *GO biological processes* | 0.013 |
| tryptophan metabolic process (GO:0006568) | *GO biological processes* | 0.013 |
| oxidoreductase activity, acting on NAD(P)H, oxygen as acceptor (GO:0050664) | *GO molecular functions* | 0.014 |
| establishment of skin barrier (GO:0061436) | *GO biological processes* | 0.015 |
| amine catabolic process (GO:0009310) | *GO biological processes* | 0.015 |
| innervation (GO:0060384) | *GO biological processes* | 0.015 |
| cellular biogenic amine catabolic process (GO:0042402) | *GO biological processes* | 0.015 |
| contractile fiber part (GO:0044449) | *GO cellular component* | 0.015 |
| 14-3-3 protein binding (GO:0071889) | *GO molecular functions* | 0.016 |
| intrinsic apoptotic signaling pathway in response to oxidative stress (GO:0008631) | *GO biological processes* | 0.017 |
| structural constituent of eye lens (GO:0005212) | *GO molecular functions* | 0.018 |
| dystrophin-associated glycoprotein complex (GO:0016010) | *GO cellular component* | 0.020 |
| intermediate filament (GO:0005882) | *GO cellular component* | 0.021 |
| costamere (GO:0043034) | *GO cellular component* | 0.025 |
| integral component of plasma membrane (GO:0005887) | *GO cellular component* | 0.028 |
| intrinsic component of plasma membrane (GO:0031226) | *GO cellular component* | 0.038 |
| terminal bouton (GO:0043195) | *GO cellular component* | 0.044 |
| trans-Golgi network membrane (GO:0032588) | *GO cellular component* | 0.048 |

Top 10 GO processes are shown where number of entries exceeds 10.
