# Supplementary material for: Alteration of Gene Expression, DNA Methylation, and Histone Methylation in Free Radical Scavenging Networks in Adult Mouse Hippocampus following Fetal Alcohol Exposure
Source: PLoS One. 2016 May 2;11(5):e0154836. doi: 10.1371/journal.pone.0154836 (PMC4852908; doi:10.1371/journal.pone.0154836)
Supplement: S2 Table — The top and bottom 20 differentially methylated regions (DMRs) according to AMS are shown with the proximal gene including distance to the gene transcriptional start site (TSS). (DOCX) [file pone.0154836.s003.docx]

**S2 Table. Top 20 increased and decreases in DNA methylation from MeDIP-chip microarray analysis.**

| **Gene Symbol** | **Chromosome** | **Distance to TSS** | **AMS difference** | ***p*-value** |
| --- | --- | --- | --- | --- |
| *Ncs1* | chr2 | -7895.5 | -24.94 | 0.0003 |
| *Hmcn1* | chr1 | 5556 | -21.43 | 0.0071 |
| *Fam32a* | chr8 | -5839.5 | -21.32 | 0.0040 |
| *Cib3* | chr8 | 1052.5 | -21.32 | 0.0040 |
| *Gpr98* | chr13 | 5462 | -20.88 | 0.0014 |
| *Myh11* | chr16 | 7062.5 | -20.88 | 0.0081 |
| *Tac1* | chr6 | -3165 | -20.65 | 0.0002 |
| *Pfkl* | chr10 | 2052.5 | -20.52 | 0.0133 |
| *Ttpa* | chr4 | -7008.5 | -20.44 | 0.0087 |
| *Il18* | chr9 | -5178.5 | -20.43 | 0.0065 |
| *Bco2* | chr9 | 5050.5 | -20.43 | 0.0065 |
| *Tex12* | chr9 | -1079.5 | -20.43 | 0.0065 |
| *Man2a1* | chr17 | -7326 | -19.82 | 0.0208 |
| *Oxsr1* | chr9 | 3808.5 | -19.50 | 0.0222 |
| *Kcnq2* | chr2 | -2858.5 | -19.32 | 0.0152 |
| *Rab17* | chr1 | 3084.5 | -19.29 | 0.0043 |
| *Rab17* | chr1 | 1131.5 | -19.29 | 0.0043 |
| *Rps6kl1* | chr12 | 1154.5 | -19.26 | 0.0204 |
| *Sik1* | chr17 | -1209.5 | -19.22 | 0.0201 |
| *Kcna10* | chr3 | -2048 | -19.19 | 0.0040 |
| *Plcd4* | chr1 | -4624 | 21.94 | 0.0182 |
| *Heatr5b* | chr17 | 6190.5 | 21.96 | 0.0025 |
| *Gnb2* | chr5 | 7852.5 | 22.13 | 0.0252 |
| *Cdkn1a* | chr17 | -3995 | 22.14 | 0.0051 |
| *Cdkn1a* | chr17 | -6781 | 22.14 | 0.0051 |
| *LOC100499420* | chr17 | 8029 | 22.14 | 0.0051 |
| *Neurog3* | chr10 | -6828.5 | 22.21 | 0.0023 |
| *Ppfia1* | chr7 | 4518.5 | 22.29 | 0.0021 |
| *3632451O06Rik* | chr14 | -823.5 | 22.40 | 0.0042 |
| *Tmem126a* | chr7 | 7698 | 22.54 | 0.0023 |
| *Mir1966* | chr8 | -384.5 | 22.57 | 0.0014 |
| *Tle4* | chr19 | 2258.5 | 22.59 | 0.0338 |
| *Map3k9* | chr12 | 5701 | 22.64 | 0.0019 |
| *Smek1* | chr12 | 5334 | 22.95 | 0.0141 |
| *Vdr* | chr15 | 5212 | 23.03 | 0.0001 |
| *Camk2n1* | chr4 | -7875.5 | 23.09 | 0.0028 |
| *Fhdc1* | chr3 | 817.5 | 23.29 | 0.0003 |
| *Gcnt2* | chr13 | 2779 | 23.69 | 0.0047 |
| *Olfr550* | chr7 | -2165 | 25.07 | 0.0061 |
| *1190005I06Rik* | chr8 | 5717.5 | 26.33 | 0.0006 |

The top and bottom 20 differentially methylated regions (DMRs) according to AMS are shown with the proximal gene including distance to the gene transcriptional start site (TSS).
