# Supplementary material for: Alteration of Gene Expression, DNA Methylation, and Histone Methylation in Free Radical Scavenging Networks in Adult Mouse Hippocampus following Fetal Alcohol Exposure
Source: PLoS One. 2016 May 2;11(5):e0154836. doi: 10.1371/journal.pone.0154836 (PMC4852908; doi:10.1371/journal.pone.0154836)
Supplement: S3 Table — Top 10 GO processes are shown where number of entries exceeds 10. (DOCX) [file pone.0154836.s004.docx]

**S3 Table. Gene ontology (GO) analysis of genes with DMRs in their promoter.**

| **GO term** | **Process** | ***p-*value** |
| --- | --- | --- |
| Basement membrane (GO:0005604) | GO cellular component | 0.0002 |
| Myeloid leukocyte differentiation (GO:0002573) | GO biological processes | 0.0004 |
| Myeloid cell differentiation (GO:0030099) | GO biological processes | 0.0004 |
| Cellular response to thyroid hormone stimulus (GO:0097067) | GO biological processes | 0.0008 |
| Extracellular matrix part (GO:0044420) | GO cellular component | 0.0008 |
| Positive regulation of cell fate commitment (GO:0010455) | GO biological processes | 0.0013 |
| Negative regulation of lipid biosynthetic process (GO:0051055) | GO biological processes | 0.0017 |
| Granulocyte differentiation (GO:0030851) | GO biological processes | 0.0020 |
| Response to thyroid hormone (GO:0097066) | GO biological processes | 0.0020 |
| Hormone receptor binding (GO:0051427) | GO molecular function | 0.0020 |
| Positive regulation of developmental growth (GO:0048639) | GO biological processes | 0.0022 |
| Cellular response to hormone stimulus (GO:0032870) | GO biological processes | 0.0026 |
| Regulation of neuron projection development (GO:0010975) | GO biological processes | 0.0027 |
| Anchored component of membrane (GO:0031225) | GO cellular component | 0.0027 |
| Estrogen receptor binding (GO:0030331) | GO molecular function | 0.0027 |
| Growth factor activity (GO:0008083) | GO molecular function | 0.0060 |
| S100 protein binding (GO:0044548) | GO molecular function | 0.0074 |
| Glycosaminoglycan binding (GO:0005539) | GO molecular function | 0.0076 |
| Extrinsic component of cytoplasmic side of plasma membrane (GO:0031234) | GO cellular component | 0.011 |
| Extracellular matrix (GO:0031012) | GO cellular component | 0.011 |
| Neuropeptide receptor binding (GO:0071855) | GO molecular function | 0.011 |
| Sequence-specific DNA binding RNA polymerase II transcription factor activity (GO:0000981) | GO molecular function | 0.013 |
| Tumor necrosis factor receptor superfamily binding (GO:0032813) | GO molecular function | 0.013 |
| FMN binding (GO:0010181) | GO molecular function | 0.015 |
| Notch binding (GO:0005112) | GO molecular function | 0.015 |
| Ruffle (GO:0001726) | GO cellular component | 0.021 |
| Cortical cytoskeleton (GO:0030863) | GO cellular component | 0.022 |
| Extrinsic component of plasma membrane (GO:0019897) | GO cellular component | 0.025 |
| Cell surface (GO:0009986) | GO cellular component | 0.027 |
| Exosome (RNase complex) (GO:0000178) | GO cellular component | 0.028 |

Top 10 GO processes are shown where number of entries exceeds 10.
