# Supplementary material for: Alteration of Gene Expression, DNA Methylation, and Histone Methylation in Free Radical Scavenging Networks in Adult Mouse Hippocampus following Fetal Alcohol Exposure
Source: PLoS One. 2016 May 2;11(5):e0154836. doi: 10.1371/journal.pone.0154836 (PMC4852908; doi:10.1371/journal.pone.0154836)
Supplement: S4 Table — The top and bottom 20 regions of differential histone methylation (RDHMs) according to MAT score are shown with the proximal gene including distance to the gene transcriptional start site (TSS). (DOCX) [file pone.0154836.s005.docx]

**S4 Table. Top 20 increases and decreases in H3K4me3 methylation from ChIP-chip microarray analysis.**

| **Gene Symbol** | **Chromosome** | **Distance to TSS** | **MAT-score** | ***p*-value** |
| --- | --- | --- | --- | --- |
| *Pcdha4-g* | chr18 | 781334 | -11.1605 | 9.36E-05 |
| *Pcdhga1* | chr18 | 72130 | -11.1605 | 9.36E-05 |
| *Pcdhga2* | chr18 | 64970 | -11.1605 | 9.36E-05 |
| *Pcdhga3* | chr18 | 59740 | -11.1605 | 9.36E-05 |
| *Pcdhgb1* | chr18 | 53617 | -11.1605 | 9.36E-05 |
| *Pcdhga4* | chr18 | 48675 | -11.1605 | 9.36E-05 |
| *Pcdhgb2* | chr18 | 44216 | -11.1605 | 9.36E-05 |
| *Pcdhga5* | chr18 | 39574 | -11.1605 | 9.36E-05 |
| *Pcdhga6* | chr18 | 26846 | -11.1605 | 9.36E-05 |
| *Pcdhga7* | chr18 | 19241 | -11.1605 | 9.36E-05 |
| *Pcdhgb4* | chr18 | 13521 | -11.1605 | 9.36E-05 |
| *Pcdhga8* | chr18 | 8369 | -11.1605 | 9.36E-05 |
| *Pcdhgb5* | chr18 | 2921 | -11.1605 | 9.36E-05 |
| *Pcdhga9* | chr18 | -2841 | -11.1605 | 9.36E-05 |
| *Pcdha4-g* | chr18 | 722971 | -8.17202 | 9.36E-05 |
| *Pcdhga1* | chr18 | 13767 | -8.17202 | 9.36E-05 |
| *Pcdhga2* | chr18 | 6607 | -8.17202 | 9.36E-05 |
| *Pcdhga3* | chr18 | 1377 | -8.17202 | 9.36E-05 |
| *Pcdhgb1* | chr18 | -4726 | -8.17202 | 9.36E-05 |
| *3110082I17Rik* | chr5 | 79747 | -7.15166 | 9.36E-05 |
| *Pou3f3os* | chr1 | -1404 | 5.41674 | 9.36E-05 |
| *Pou3f3* | chr1 | -896 | 5.41674 | 9.36E-05 |
| *2900092D14Rik* | chr1 | -4086 | 5.41674 | 9.36E-05 |
| *Olfr406* | chr11 | -2515 | 5.42421 | 9.36E-05 |
| *Serpinf2* | chr11 | -2463 | 5.45151 | 9.36E-05 |
| *Wdr81* | chr11 | 12733 | 5.45151 | 9.36E-05 |
| *Olfr391-ps* | chr11 | -2489 | 5.50726 | 9.36E-05 |
| *Olfr1370* | chr13 | -2489 | 5.55823 | 9.36E-05 |
| *Efcab6* | chr15 | 112908 | 5.61754 | 9.36E-05 |
| *Cyp2j12* | chr4 | -2379 | 5.7489 | 9.36E-05 |
| *1700109G14Rik* | chr14 | -1907 | 6.00292 | 9.36E-05 |
| *Arl11* | chr14 | -2491 | 6.00292 | 9.36E-05 |
| *Akap1* | chr11 | -2480 | 6.03939 | 9.36E-05 |
| *Flii* | chr11 | -577 | 6.21834 | 9.36E-05 |
| *Mief2* | chr11 | -579 | 6.21834 | 9.36E-05 |
| *Mir5100* | chr11 | -844 | 6.21834 | 9.36E-05 |
| *Tspan8* | chr10 | -2378 | 6.26893 | 9.36E-05 |
| *Mid1* | chrX | 294406 | 7.19251 | 9.36E-05 |
| *G530011O06Rik* | chrX | -687 | 7.19251 | 9.36E-05 |
| *Olfr284* | chr15 | -2489 | 7.1997 | 9.36E-05 |

The top and bottom 20 regions of differential histone methylation (RDHMs) according to MAT score are shown with the proximal gene including distance to the transcriptional start site (TSS).
