# Supplementary material for: Alteration of Gene Expression, DNA Methylation, and Histone Methylation in Free Radical Scavenging Networks in Adult Mouse Hippocampus following Fetal Alcohol Exposure
Source: PLoS One. 2016 May 2;11(5):e0154836. doi: 10.1371/journal.pone.0154836 (PMC4852908; doi:10.1371/journal.pone.0154836)
Supplement: S5 Table — The top and bottom 20 regions of differential histone methylation (RDHMs) according to MAT score are shown with the proximal gene including distance to the transcriptional start site (TSS). (DOCX) [file pone.0154836.s006.docx]

**S5 Table. Top 20 increased and decreases in H3K27me3 methylation from ChIP-chip microarray analysis.**

| Gene Symbol | chromosome | Distance to TSS | MAT-score | p-value |
| --- | --- | --- | --- | --- |
| *Hoxd10* | chr2 | 1384 | -22.44 | 9.36E-05 |
| *Hoxd9* | chr2 | -4468 | -22.44 | 9.36E-05 |
| *Hoxa7* | chr6 | -2823 | -19.74 | 9.36E-05 |
| *Mir337* | chr12 | -2265 | -18.38 | 9.36E-05 |
| *Mir3544* | chr12 | 2350 | -18.38 | 9.36E-05 |
| *Mir540* | chr12 | -2556 | -18.38 | 9.36E-05 |
| *Mir665* | chr12 | -2790 | -18.38 | 9.36E-05 |
| *Mir3070a* | chr12 | -4419 | -18.38 | 9.36E-05 |
| *G6b* | chr17 | -1723 | -16.41 | 0.00019 |
| *Ly6g6c* | chr17 | 583 | -16.41 | 0.00019 |
| *Hoxb9* | chr11 | -1416 | -15.79 | 0.00019 |
| *8030411F24Rik* | chr2 | 3111 | -15.00 | 0.00028 |
| *Cst12* | chr2 | -4221 | -15.00 | 0.00028 |
| *Bcl6b* | chr11 | -2999 | -14.85 | 0.00028 |
| *Mir497b* | chr11 | 1999 | -14.85 | 0.00028 |
| *Mir497* | chr11 | -1899 | -14.85 | 0.00028 |
| *Mir195* | chr11 | -2224 | -14.85 | 0.00028 |
| *Prss45* | chr9 | 5230 | -14.77 | 0.00028 |
| *Prss46* | chr9 | -4668 | -14.77 | 0.00028 |
| *Mir666* | chr12 | 1832 | -14.73 | 0.00028 |
| *Txndc9* | chr1 | -389 | 4.20 | 0.00019 |
| *Eif5b* | chr1 | -392 | 4.20 | 0.00019 |
| *Pnkd* | chr1 | 6398 | 4.25 | 0.00019 |
| *Tmbim1* | chr1 | 12885 | 4.25 | 0.00019 |
| *S1pr2* | chr9 | -2437 | 4.29 | 0.00019 |
| *Gm17296* | chr8 | -2489 | 4.30 | 0.00019 |
| *Zfhx3* | chr8 | 232602 | 4.31 | 0.00019 |
| *Dnm1* | chr2 | 42281 | 4.34 | 0.00019 |
| *Itm2b* | chr14 | -2479 | 4.35 | 0.00019 |
| *Snord116* | chr7 | -2489 | 4.36 | 0.00019 |
| *Snord116l1* | chr7 | -2489 | 4.36 | 0.00019 |
| *Zfp648* | chr1 | 3990 | 4.37 | 0.00019 |
| *Flii* | chr11 | -577 | 4.42 | 0.00019 |
| *Mief2* | chr11 | -579 | 4.42 | 0.00019 |
| *Mir5100* | chr11 | -844 | 4.42 | 0.00019 |
| *Lrch1* | chr14 | -2489 | 4.43 | 0.00019 |
| *Lrch1* | chr14 | -2489 | 4.43 | 0.00019 |
| *Ctnnd2* | chr15 | 474291 | 4.99 | 9.36E-05 |
| *Pou3f1* | chr4 | -2461 | 5.40 | 9.36E-05 |
| *Sox9* | chr11 | -2363 | 6.98 | 9.36E-05 |

The top and bottom 20 regions of differential histone methylation (RDHMs) according to MAT score are shown with the proximal gene including distance to the gene transcriptional start site (TSS).
