# Supplementary material for: Alteration of Gene Expression, DNA Methylation, and Histone Methylation in Free Radical Scavenging Networks in Adult Mouse Hippocampus following Fetal Alcohol Exposure
Source: PLoS One. 2016 May 2;11(5):e0154836. doi: 10.1371/journal.pone.0154836 (PMC4852908; doi:10.1371/journal.pone.0154836)
Supplement: S6 Table — Top 10 GO processes are shown where number of entries exceeds 10. (DOCX) [file pone.0154836.s007.docx]

**S6 Table. Gene ontology (GO) analysis of genes with H3K4me3 RDHMs in their promoter.**

| GO term | Process | *p-*value |
| --- | --- | --- |
| cell-cell adhesion via plasma-membrane adhesion molecules (GO:0098742) | GO biological process | 1.49E-09 |
| cell-cell adhesion (GO:0098609) | GO biological process | 1.66E-09 |
| calcium ion binding (GO:0005509) | GO molecular function | 0.00012 |
| phosphoprotein binding (GO:0051219) | GO molecular function | 0.00091 |
| ionotropic glutamate receptor complex (GO:0008328) | GO cellular component | 0.0020 |
| nervous system development (GO:0007399) | GO biological process | 0.0030 |
| negative regulation of growth (GO:0045926) | GO biological process | 0.0033 |
| regulation of neuron differentiation (GO:0045664) | GO biological process | 0.0036 |
| sympathetic nervous system development (GO:0048485) | GO biological process | 0.0042 |
| regulation of stress-activated MAPK cascade (GO:0032872) | GO biological process | 0.0042 |
| cellular response to organonitrogen compound (GO:0071417) | GO biological process | 0.0043 |
| regulation of stress-activated protein kinase signaling cascade (GO:0070302) | GO biological process | 0.0044 |
| spectrin-associated cytoskeleton (GO:0014731) | GO cellular component | 0.0045 |
| synaptic membrane (GO:0097060) | GO cellular component | 0.0056 |
| receptor signaling complex scaffold activity (GO:0030159) | GO molecular function | 0.0070 |
| postsynaptic membrane (GO:0045211) | GO cellular component | 0.0078 |
| protein phosphorylated amino acid binding (GO:0045309) | GO molecular function | 0.0081 |
| vinculin binding (GO:0017166) | GO molecular function | 0.011 |
| transcription factor complex (GO:0005667) | GO cellular component | 0.011 |
| Ras GTPase binding (GO:0017016) | GO molecular function | 0.011 |
| cell body (GO:0044297) | GO cellular component | 0.012 |
| SH3 domain binding (GO:0017124) | GO molecular function | 0.013 |
| Rab GTPase binding (GO:0017137) | GO molecular function | 0.013 |
| gamma-catenin binding (GO:0045295) | GO molecular function | 0.013 |
| GTPase binding (GO:0051020) | GO molecular function | 0.015 |
| neuronal cell body (GO:0043025) | GO cellular component | 0.023 |
| axon (GO:0030424) | GO cellular component | 0.023 |
| cytosol (GO:0005829) | GO cellular component | 0.027 |
| synapse part (GO:0044456) | GO cellular component | 0.028 |

Top 10 GO processes are shown where number of entries exceeds 10.
