# Supplementary material for: Alteration of Gene Expression, DNA Methylation, and Histone Methylation in Free Radical Scavenging Networks in Adult Mouse Hippocampus following Fetal Alcohol Exposure
Source: PLoS One. 2016 May 2;11(5):e0154836. doi: 10.1371/journal.pone.0154836 (PMC4852908; doi:10.1371/journal.pone.0154836)
Supplement: S7 Table — Top 10 GO processes are shown where number of entries exceeds 10. (DOCX) [file pone.0154836.s008.docx]

**S7 Table. Gene ontology (GO) analysis of genes with H3K27me3 RDHMs in their promoter.**

| GO term | Process | *p-*value |
| --- | --- | --- |
| homophilic cell adhesion via plasma membrane adhesion molecules (GO:0007156) | GO biological process | 9.48E-10 |
| cell-cell adhesion via plasma-membrane adhesion molecules (GO:0098742) | GO biological process | 2.52E-08 |
| cell-cell adhesion (GO:0098609) | GO biological process | 2.67E-08 |
| mammary gland development (GO:0030879) | GO biological process | 6.15E-05 |
| calcium ion binding (GO:0005509) | GO molecular function | 0.00011 |
| neuron fate specification (GO:0048665) | GO biological process | 0.0015 |
| proximal/distal pattern formation (GO:0009954) | GO biological process | 0.0018 |
| microfibril (GO:0001527) | GO cellular component | 0.0032 |
| negative regulation of cell aging (GO:0090344) | GO biological process | 0.0056 |
| fibril (GO:0043205) | GO cellular component | 0.0060 |
| pre-mRNA binding (GO:0036002) | GO molecular function | 0.0071 |
| insulin-like growth factor receptor signaling pathway (GO:0048009) | GO biological process | 0.0084 |
| fibril organization (GO:0097435) | GO biological process | 0.0084 |
| mitochondrial calcium ion homeostasis (GO:0051560) | GO biological process | 0.0095 |
| intermediate filament cytoskeleton (GO:0045111) | GO cellular component | 0.011 |
| ankyrin binding (GO:0030506) | GO molecular function | 0.016 |
| extracellular matrix structural constituent (GO:0005201) | GO molecular function | 0.023 |
| core promoter sequence-specific DNA binding (GO:0001046) | GO molecular function | 0.025 |
| integral component of mitochondrial membrane (GO:0032592) | GO cellular component | 0.038 |
| galactosyltransferase activity (GO:0008378) | GO molecular function | 0.038 |
| GOlgi apparatus (GO:0005794) | GO cellular component | 0.043 |
| oxygen binding (GO:0019825) | GO molecular function | 0.044 |
| RNA polymerase II core promoter sequence-specific DNA binding (GO:0000979) | GO molecular function | 0.057 |
| acetylglucosaminyltransferase activity (GO:0008375) | GO molecular function | 0.059 |
| platelet alpha granule lumen (GO:0031093) | GO cellular component | 0.059 |
| serine-type endopeptidase activity (GO:0004252) | GO molecular function | 0.059 |
| transcription factor complex (GO:0005667) | GO cellular component | 0.064 |
| MLL5-L complex (GO:0070688) | GO cellular component | 0.069 |
| membrane coat (GO:0030117) | GO cellular component | 0.074 |
| hemidesmosome (GO:0030056) | GO cellular component | 0.076 |

Top 10 GO processes are shown where number of entries exceeds 10.
