# Supplementary material for: Alteration of Gene Expression, DNA Methylation, and Histone Methylation in Free Radical Scavenging Networks in Adult Mouse Hippocampus following Fetal Alcohol Exposure
Source: PLoS One. 2016 May 2;11(5):e0154836. doi: 10.1371/journal.pone.0154836 (PMC4852908; doi:10.1371/journal.pone.0154836)
Supplement: S8 Table — Top 10 GO processes are shown for each. (DOCX) [file pone.0154836.s009.docx]

**S8 Table. Gene ontology (GO) analysis of genes with either a DMR or RDHM in their promoter.**

| GO term | Process | *p-*value |
| --- | --- | --- |
| homophilic cell adhesion via plasma membrane adhesion molecules (GO:0007156) | GO biological processes | 4.43E-08 |
| cell-cell adhesion via plasma-membrane adhesion molecules (GO:0098742) | GO biological processes | 5.26E-06 |
| cell-cell adhesion (GO:0098609) | GO biological processes | 5.81E-06 |
| cell-cell adhesion (GO:0098609) | GO biological processes | 5.81E-06 |
| regulation of neuron differentiation (GO:0045664) | GO biological processes | 5.63E-05 |
| regulation of neuron projection development (GO:0010975) | GO biological processes | 0.00012 |
| myeloid cell differentiation (GO:0030099) | GO biological processes | 0.00034 |
| regulation of cell projection organization (GO:0031344) | GO biological processes | 0.00047 |
| erythrocyte differentiation (GO:0030218) | GO biological processes | 0.00050 |
| nervous system development (GO:0007399) | GO biological processes | 0.00051 |
| calcium ion binding (GO:0005509) | GO molecular functions | 0.00061 |
| basement membrane (GO:0005604) | GO cellular component | 0.0019 |
| estrogen receptor binding (GO:0030331) | GO molecular functions | 0.0021 |
| extracellular matrix part (GO:0044420) | GO cellular component | 0.0026 |
| transcription factor complex (GO:0005667) | GO cellular component | 0.0033 |
| calmodulin binding (GO:0005516) | GO molecular functions | 0.0047 |
| synapse (GO:0045202) | GO cellular component | 0.0050 |
| growth factor activity (GO:0008083) | GO molecular functions | 0.0058 |
| extracellular matrix (GO:0031012) | GO cellular component | 0.0092 |
| integrin binding (GO:0005178) | GO molecular functions | 0.010 |
| ionotropic glutamate receptor complex (GO:0008328) | GO cellular component | 0.011 |
| protein tyrosine kinase activity (GO:0004713) | GO molecular functions | 0.011 |
| axon (GO:0030424) | GO cellular component | 0.012 |
| S100 protein binding (GO:0044548) | GO molecular functions | 0.015 |
| extracellular matrix structural constituent (GO:0005201) | GO molecular functions | 0.015 |
| hormone receptor binding (GO:0051427) | GO molecular functions | 0.016 |
| gamma-catenin binding (GO:0045295) | GO molecular functions | 0.019 |
| STAGA complex (GO:0030914) | GO cellular component | 0.020 |
| synaptic membrane (GO:0097060) | GO cellular component | 0.021 |
| ruffle (GO:0001726) | GO cellular component | 0.027 |

Top 10 GO processes are shown for each.
