# Supplementary material for: Alteration of Gene Expression, DNA Methylation, and Histone Methylation in Free Radical Scavenging Networks in Adult Mouse Hippocampus following Fetal Alcohol Exposure
Source: PLoS One. 2016 May 2;11(5):e0154836. doi: 10.1371/journal.pone.0154836 (PMC4852908; doi:10.1371/journal.pone.0154836)
Supplement: S9 Table — Concentrations for each gene of interest (GOI) and the reference gene Tata-Binding Protein (TBP) are shown for each experiment. The mRNA concentration is presented as an average of the concentration of each of seven replicates in each group. The standard error of the mean (SEM) is also presented. Each replicate was also calculated as an average of three separate technical replicates. (DOCX) [file pone.0154836.s010.docx]

**S9 Table. Absolute concentrations of mRNA species from droplet digital PCR (ddPCR).**

| **Experiment** | **Target** | **Group** | **Concentration** | **SEM** |
| --- | --- | --- | --- | --- |
| *Casp3* | GOI | Control | 110.8 | 10.5 |
|  | TBP | Control | 258.8 | 28.9 |
|  | GOI | Ethanol | 170.9 | 52.8 |
|  | TBP | Ethanol | 383.7 | 23.6 |
| *Defb4* | GOI | Control | 0 | 0 |
|  | TBP | Control | 32.0 | 2.7 |
|  | GOI | Ethanol | 0.0 | 0.0 |
|  | TBP | Ethanol | 34.3 | 5.1 |
| *Krt8* | GOI | Control | 1.0 | 0.2 |
|  | TBP | Control | 16.0 | 1.8 |
|  | GOI | Ethanol | 5.1 | 2.2 |
|  | TBP | Ethanol | 24.2 | 11.5 |
| *L3mbtl4* | GOI | Control | 1.9 | 0.6 |
|  | TBP | Control | 47.1 | 7.2 |
|  | GOI | Ethanol | 1.2 | 0.3 |
|  | TBP | Ethanol | 67.1 | 28.2 |
| *Mafg* | GOI | Control | 239.9 | 26.2 |
|  | TBP | Control | 173.9 | 17.0 |
|  | GOI | Ethanol | 332.1 | 50.5 |
|  | TBP | Ethanol | 238.5 | 37.0 |
| *Stac* | GOI | Control | 17.6 | 3.5 |
|  | TBP | Control | 276.6 | 31.9 |
|  | GOI | Ethanol | 12.4 | 2.5 |
|  | TBP | Ethanol | 226.2 | 45.3 |
| *Synpo2* | GOI | Control | 3.9 | 0.5 |
|  | TBP | Control | 29.1 | 3.8 |
|  | GOI | Ethanol | 12.9 | 4.1 |
|  | TBP | Ethanol | 41.3 | 21.6 |
| *Tcf7l2* | GOI | Control | 102.0 | 16.2 |
|  | TBP | Control | 177.6 | 23.7 |
|  | GOI | Ethanol | 362.5 | 172.1 |
|  | TBP | Ethanol | 214.9 | 47.3 |
| *Tmem79* | GOI | Control | 4.4 | 1.7 |
|  | TBP | Control | 14.7 | 1.8 |
|  | GOI | Ethanol | 3.2 | 1.4 |
|  | TBP | Ethanol | 18.6 | 10.4 |
| *Vipr2* | GOI | Control | 2.5 | 0.4 |
|  | TBP | Control | 84.0 | 6.8 |
|  | GOI | Ethanol | 8.3 | 3.5 |
|  | TBP | Ethanol | 96.1 | 44.6 |

Concentrations for each gene of interest (GOI) and the reference gene tata-binding protein (TBP) are shown for each experiment. The mRNA concentration is presented as an average of the concentration of each of seven replicates in each group. The standard error of the mean (SEM) is also presented. Each replicate was also calculated as an average of three separate technical replicates.
