# Supplementary material for: Alteration of Gene Expression, DNA Methylation, and Histone Methylation in Free Radical Scavenging Networks in Adult Mouse Hippocampus following Fetal Alcohol Exposure
Source: PLoS One. 2016 May 2;11(5):e0154836. doi: 10.1371/journal.pone.0154836 (PMC4852908; doi:10.1371/journal.pone.0154836)
Supplement: S10 Table — Pyrosequencing was performed on DNA mixing controls of known methylation percentage were sequenced for each SNP. The r2 coefficient for each SNP is shown. (DOCX) [file pone.0154836.s011.docx]

**S10 Table. Pyrosequencing mixing control for cytosines of interest.**

| **Mixing control (%)** | **Percent methylation** | |
| --- | --- | --- |
|  | Acaa1 (chr9: 119342352) | Pex6 (Chr17: 4670666) |
| 0 | 5.65 | 0.00 |
| 5 | 6.90 | 7.28 |
| 10 | 11.56 | 12.41 |
| 25 | 29.75 | 30.59 |
| 50 | 47.75 | 62.67 |
| 75 | 68.67 | 80.33 |
| 100 | 90.20 | 100.00 |
| **r^2^** | 0.9967 | 0.9875 |

Pyrosequencing was performed on DNA mixing controls of known methylation percentage were sequenced for each SNP. The r^2^ coefficient for each SNP is shown.
