# Supplementary material for: Alteration of Gene Expression, DNA Methylation, and Histone Methylation in Free Radical Scavenging Networks in Adult Mouse Hippocampus following Fetal Alcohol Exposure
Source: PLoS One. 2016 May 2;11(5):e0154836. doi: 10.1371/journal.pone.0154836 (PMC4852908; doi:10.1371/journal.pone.0154836)
Supplement: S11 Table — Threshold cycle (Ct) values for each primer pair for each sample are shown. (DOCX) [file pone.0154836.s012.docx]

**S11 Table. Real-time PCR assessment of MeDIP fold enrichment.**

| Sample | Primer pair | Input DNA (Ct) | MeDIP DNA (Ct) | % (MeDIP/ Input) | Negative control (IgG)(Ct) | % (Negative (control/ Input) | Fold Enrichment |
| --- | --- | --- | --- | --- | --- | --- | --- |
| C5.3 | TSH2B | 21.060 | 22.586 | 6.944 | 38.248 | 1.34E-04 | 5.19E+04 |
|  | GAPDH | 21.628 | NA | NA | NA | NA | ~ |
| C10.5 | TSH2B | 21.874 | 23.003 | 9.148 | 33.622 | 5.82E-03 | 1.57E+03 |
|  | GAPDH | 21.737 | NA | NA | NA | NA | ~ |
| C11.2 | TSH2B | 21.129 | 22.969 | 5.584 | 37.193 | 2.92E-04 | 1.91E+04 |
|  | GAPDH | 21.570 | NA | NA | NA | NA | ~ |
| E5.1 | TSH2B | 21.185 | 23.265 | 4.731 | NA | NA | ~ |
|  | GAPDH | 21.562 | NA | NA | NA | NA | ~ |
| E10.3 | TSH2B | 21.200 | 22.763 | 6.767 | NA | NA | ~ |
|  | GAPDH | 21.426 | NA | NA | NA | NA | ~ |
| E11.1 | TSH2B | 20.524 | 22.417 | 5.387 | 36.185 | 3.86E-04 | 1.39E+04 |
|  | GAPDH | 21.117 | NA | NA | NA | NA | ~ |

Threshold cycle (Ct) values for each primer pair for each sample are shown.
